# WDR72 Is Required for Urinary Acidification and Normal H^+^‐ATPase Activity in Intercalated Cells in Mice

**DOI:** 10.1111/apha.70165

**Published:** 2026-02-04

**Authors:** Hannah Auwerx, Moana Busch‐Dohr, Xiaoxu Li, Carsten A. Wagner, Soline Bourgeois

**Affiliations:** ^1^ Institute of Physiology University of Zurich Zurich Switzerland; ^2^ Laboratory of Integrative Systems Physiology École Polytechnique Fédérale de Lausanne (EPFL) Lausanne Switzerland

**Keywords:** acid–base homeostasis, distal renal tubular acidosis, intercalated cells, sex difference, WD40 repeat domain protein

## Abstract

**Aim:**

Biallelic inactivating *WDR72* variants are linked to distal renal tubular acidosis (dRTA), nephrocalcinosis, and amelogenesis imperfecta. The kidney shows high *WDR72* expression; its precise localization and function remain unclear. WDR72 is a member of the WD40 repeat domain protein family—a large group of scaffold proteins involved in various pathways, including vesicular trafficking—which has been suggested as a potential role for WDR72. This study investigates WDR72 expression and its role in renal acid–base homeostasis.

**Methods:**

We analyzed *WDR72/Wdr72* expression in single‐cell transcriptome data from human and murine kidneys. We characterized *Wdr72* ^*−/−*^ female and male mice and assessed *Wdr72* mRNA and protein localization, the ability of the kidney to excrete acid, and the expression and function of the H^+^‐ATPase.

**Results:**

Transcriptome data showed that *WDR72/Wdr72* is highly expressed in intercalated cells and other nephron segments. Immunohistochemistry localized WDR72 mostly at the apical membrane of type A‐intercalated cells (A‐IC). *Wdr72* ^*−/−*^ mice exhibited alkaline urine under normal conditions, but only female knockout mice developed a pronounced metabolic acidosis upon dietary acid loading. Western blot analyses revealed sex‐dependent WDR72 expression changes with acid loading. Expression of several H^+^‐ATPase subunits was dysregulated in *Wdr72* ^*−/−*^ kidneys while their localization in intercalated cells remained intact. Lower expression of H^+^‐ATPase subunits was paralleled by reduced H^+^‐ATPase activity observed in isolated microperfused collecting ducts.

**Conclusion:**

These findings identify WDR72 as a critical regulator of type A‐intercalated cell dependent urinary acidification, modulating H^+^‐ATPase activity. The sex‐specific metabolic phenotype reveals a novel mechanism underlying sex differences in renal acid handling.

## 1 Introduction

Biallelic variants in WDR72 have been identified in individuals with autosomal recessive amelogenesis imperfecta [1, 2, 3, 4]—a disorder of enamel development [5]—and in patients with distal renal tubular acidosis (dRTA) [4, 6, 7, 8], a condition characterized by impaired urinary acidification by the distal nephron, causing metabolic acidosis [9]. Furthermore, genome‐wide association studies (GWAS) associated *WDR72* variants with urinary pH, urinary uromodulin levels, renal calcifications, and the risk to develop chronic kidney disease (CKD), underlining a broader role in renal physiology and pathophysiology [10, 11, 12, 13, 14, 15].

WDR72 (WD repeat‐containing protein 72) is a member of the WD40 repeat protein family, a group of evolutionarily conserved proteins characterized by their role in facilitating protein–protein interactions. WD40 repeat proteins typically serve as scaffolding units, assembling and stabilizing multiprotein complexes involved in a wide array of cellular processes. Despite the well‐documented general functions of several WD40 proteins [16], the precise biological role of WDR72 remains poorly understood.

WDR72 shares approximately 40% sequence homology with WDR7, a known component of the rabconnectin‐3 complex, acting together with the protein DMXL1 [17, 18]. In yeast, this multiprotein scaffold complex is involved in regulating the acidification of intracellular organelles such as lysosomes and endosomes through its interaction with the vacuolar H^+^‐ATPase (V‐ATPase) proton pump [19, 20]. Although WDR72 has not been directly linked to the rabconnectin‐3 complex, its homology with WDR7 suggests that it may participate in similar cellular mechanisms—namely, pH regulation and vesicular trafficking [18].

The WDR72 protein has been detected in AQP2‐positive tubules of the mouse kidney, a nephron segment that contains both AQP2‐expressing principal cells and type A and B intercalated cells (IC). However, the exact cellular localization of WDR72 within this region remains unclear [12]. Intercalated cells play a crucial role in maintaining systemic acid–base balance by regulating the secretion of protons and bicarbonate. The restricted expression of WDR72 to this acid‐secreting portion of the distal nephron aligns with the clinical features observed in dRTA.

Multimeric vacuolar H^+^‐ATPases are critical for acid secretion and urinary acidification by intercalated cells as evident from loss‐of‐function genetic variants in the a4 (ATP6V0A4) or B1 (ATP6V1B1) subunits in patients with dRTA [9, 21]. In the kidney, WDR7, a WDR72 paralog, is part of the interactome of the multimeric H^+^‐ATPases through interactions with the B1 subunit [22], raising the possibility that WDR72 may also participate in the same regulatory network.

Here, we characterized the expression and localization of *Wdr72* mRNA and WDR72 protein in the mouse kidney using in situ RNA hybridization and immunohistochemistry. Furthermore, we demonstrate that *Wdr72‐*deficient female mice exhibit a severe incomplete metabolic acidosis phenotype upon acid loading, characterized by impaired ammonium excretion and decreased blood bicarbonate and pH. Finally, we provide evidence that WDR72 plays a key role in modulating H^+^‐ATPase expression and function. These results explain the development of dRTA in patients with genetic variants in *WDR72*, highlight WDR72 as a critical regulator of renal acid–base balance, and suggest potential sex‐specific differences in its physiological role.

## 2 Results

### 2.1 *Wdr72* mRNA Is Expressed in All Tubular Cells Whereas WDR72 Protein Is Observed Only in Intercalated Cells in the Mouse Kidney

**FIGURE 1:**
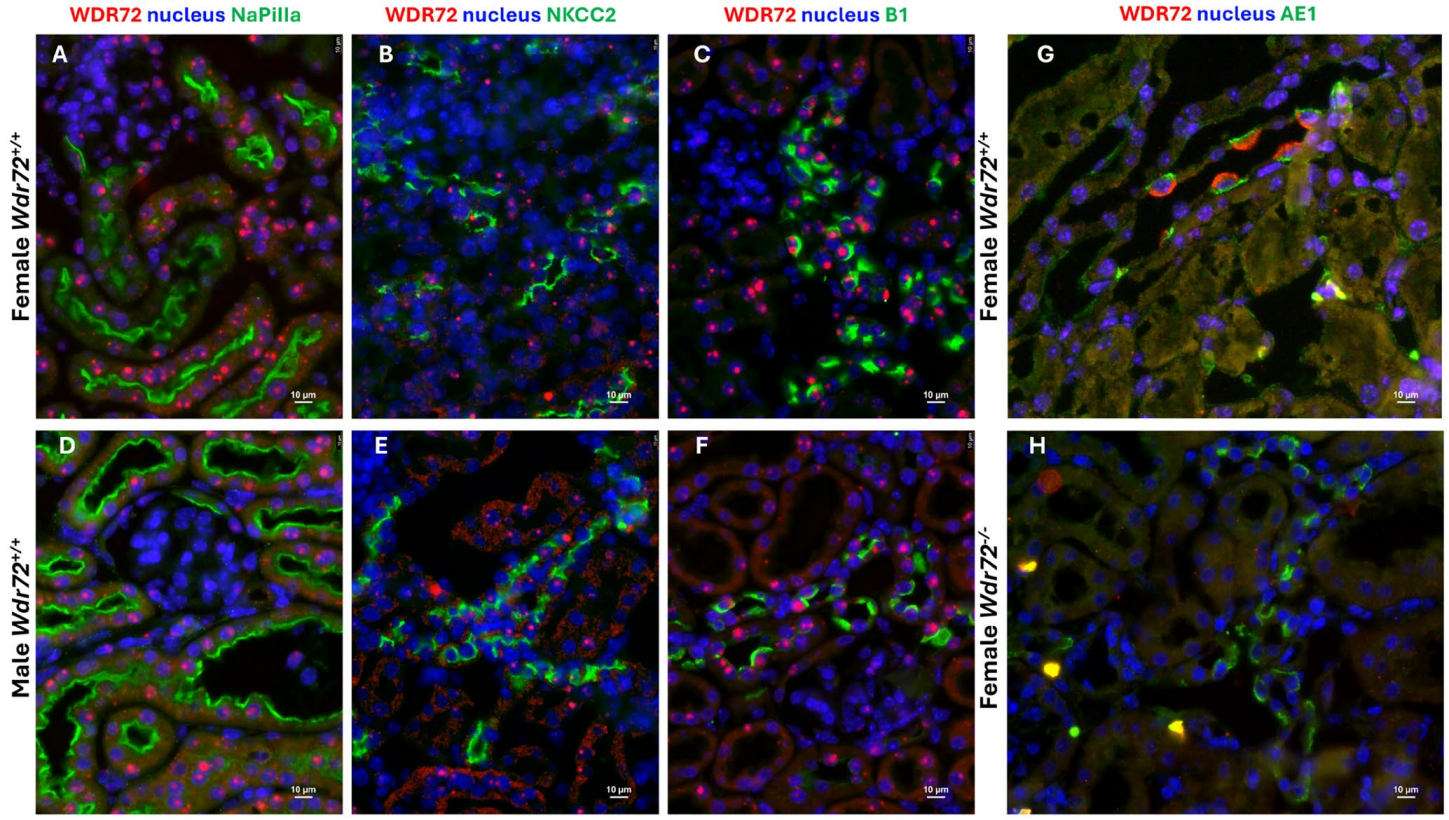
Expression patterns and cellular localization of *Wdr72* mRNA and WDR72 protein in mouse kidney cells. (A–F) Kidney sections from female and male mice receiving an HCl diet for 4 days were co‐stained with antibodies against; (A and D) NaPiIIa (green), (B and E) NKCC2 (green) and (C and F) *Wdr72* mRNA probe (red). Nuclei were marked in blue using DAPI. *Wdr72* mRNA was observed in all nephron segments with no difference between sexes. (G and H) Kidney sections from female *Wdr72* ^*+/+*^ and *Wdr72* ^*−/−*^ mice treated with a 4 days HCl diet were co‐stained with antibodies against AE1 (green) and WDR72 (red) together with DAPI in blue. WDR72 was expressed only in kidneys from *Wdr72* ^*+/+*^ mice and exclusively at the luminal side of AE1 positive cells. Original magnification 400×.

We determined the localization of *Wdr72* by performing in situ mRNA detection and WDR72 protein immunolocalization on female and male mouse kidney tissues after 4 days of acid loading (Figure 1 and Figures S1 and S2). To confirm the identity of the different nephron segments, we co‐stained *Wdr72 m*RNA with NaPiIIa protein for the proximal tubule, NKCC2 protein for the thick ascending limb, and B1, a subunit of the H^+^‐ATPase protein, for intercalated cells of the collecting duct. We observed that *Wdr72* mRNA is expressed at low levels in glomeruli but was detectable in proximal tubules, thick ascending limbs of the loop of Henle, and all intercalated cells, with no apparent difference in expression between sexes (Figure 1A–F). The specificity of the *Wdr72* probe was confirmed by the absence of staining in kidney tissue from *Wdr72* ^−/−^ mice (Figure S1).

**FIGURE 2:**
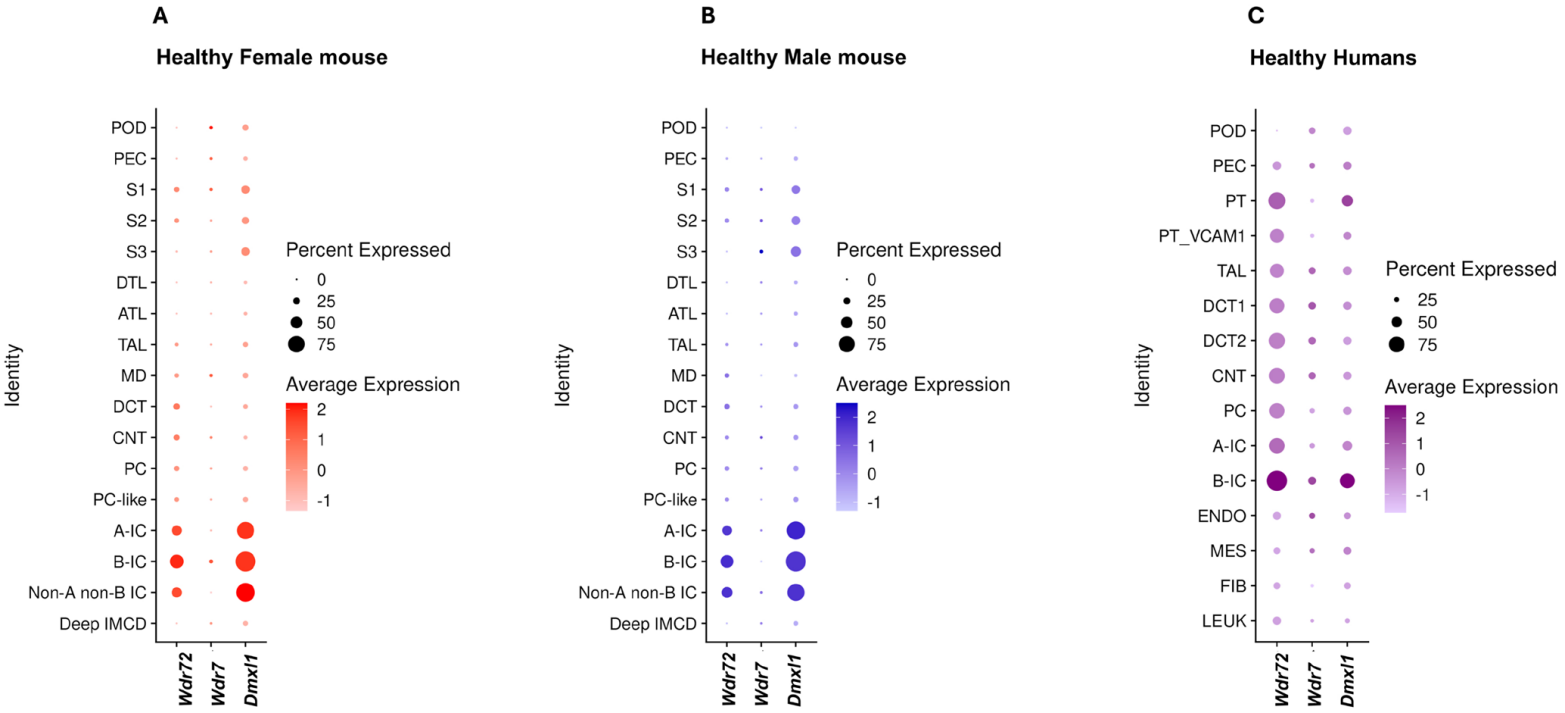
Expression of *WDR72/Wdr72*, *WDR7/Wdr7*, and *DMXL1/Dmlx1* mRNA in single kidney cells from humans and mice. Single‐nuclei/cell RNA‐seq expression of *WDR72/Wdr72*, *WDR7/Wdr7*, and *DMXL1/Dmlx1* along the nephron in (A) Healthy Female mouse, (B) Healthy Male mouse (GSE129798), and (C) Healthy Human (GSE151302). Cell type abbreviations: Mouse—POD, PEC, S1–S3: Proximal Tubule Segments 1–3, DTL, ATL, TAL, MD: Macula Densa, DCT, CNT, PC, PC‐like: Principal‐like Cell, A‐IC/B‐IC/Non‐A non‐B IC: Intercalated Cell types, Deep IMCD: Deep Inner Medullary Collecting Duct. Human—A‐IC, A‐intercalated cell; B‐IC, B‐intercalated cell; CNT, connecting tubule; DCT, distal convoluted tubule; ENDO, endothelial cell; FIB, fibroblast; LEUK, leukocyte.; MES, mesangial cell; PC, principal cell; PEC, parietal epithelial cell; POD, podocyte; PT, proximal tubule; PT_VCAM1, proximal tubule with VCAM1 expression; TAL, thick ascending limb.

WDR72 protein was colocalized with the anion exchanger 1 (AE1), a marker of type A intercalated cells. We observed a strict localization of WDR72 at the apical side of type A‐IC and no specific detectable staining in other kidney structures (Figure 1G). Moreover, WDR72 protein was also found to localize to type B intercalated cells marked by the presence of pendrin (Figure S3). The specificity of the anti‐WDR72 antibody was confirmed on *Wdr72* ^*−/−*^ kidney tissue and showed no staining in IC (Figure 1H). Finally, we compared our data with published single‐cell RNA‐sequencing datasets of healthy kidneys in female and male mice, as well as healthy humans, which confirmed our findings (Figure 2) [23, 24]. In mice, *Wdr72* showed its highest expression in intercalated cells, with a marked enrichment in type B intercalated cells. The rabconnectin‐3 protein *Dmxl1* displayed a very similar expression pattern in both mouse and human kidneys. By contrast, *Wdr7* expression remained uniformly low across the nephron, with no region showing significant enrichment (Figure 2A–C). No sex‐related differences in expression were detected in mouse kidney (Figure 2A,B). In humans, *WDR72* expression appeared more broadly distributed across multiple kidney cell types; however, consistent with the mouse data, its strongest expression was again detected in intercalated cells, particularly in type B intercalated cells (Figure 2C).

### 2.2 Absence of *Wdr72* Causes Incomplete dRTA in Female Mice With a Milder Phenotype in Male Mice

**FIGURE 3:**
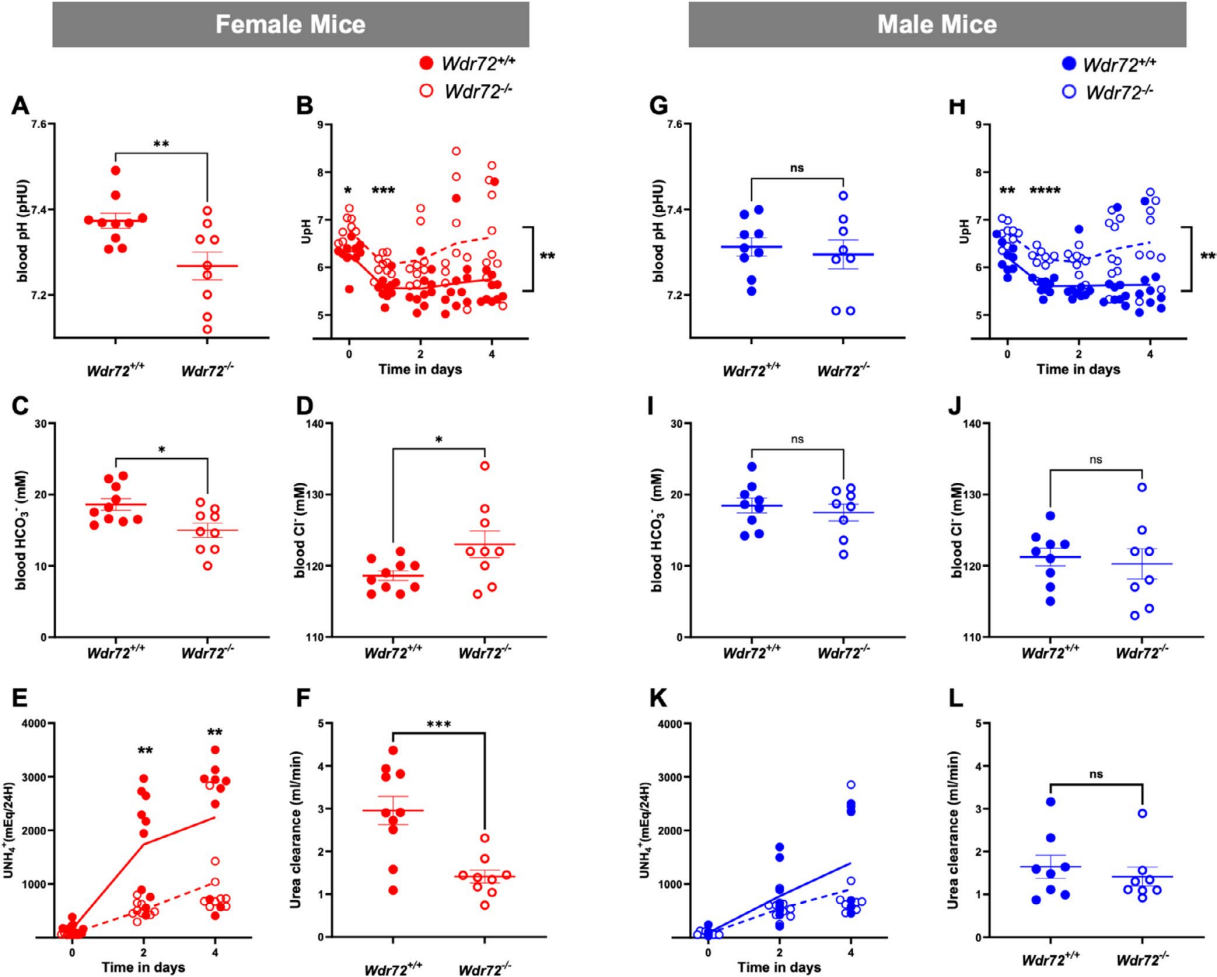
Acid loading causes more pronounced acidosis in female *Wdr72* ^*−/−*^ mice. Following 4 days of HCl loading, female mice (red dots: Filled for *Wdr72* ^*+/+*^ and empty for *Wdr72* ^*−/−*^) showed (A) a reduction in blood pH, (B) an inappropriately alkaline urine, (C) decreased bicarbonate levels, and (D) hypochloremia. (E) Urinary ammonium (NH_4_^+^) excretion was significantly reduced compared to their littermates, along with (F) a decline in urea clearance. In contrast, their male *Wdr72* ^*−/−*^ littermates (blue dots) displayed a milder phenotype, characterized solely by (G–L) a persistently more alkaline urine during the acid challenge. Values are means ± SEM. Statistical significance was assessed by unpaired *t*‐test or 2‐way ANOVA. *p ≤ 0.05, **p ≤ 0.01, ***p ≤ 0.001, ****p ≤ 0.0001.

At baseline, *Wdr72* ^*−/−*^ female and male mice fed a standard laboratory diet exhibited more alkaline urine compared to their wildtype (WT) littermates, with no other differences in urine or blood parameters measured (Tables S1–S3). Next, we studied the ability of *Wdr72* ^*−/−*^ mice to respond to an acid load. Both female and male mice were fed for 4 days a HCl diet inducing a chronic acid load [25, 26, 27, 28]. Figure 3 and Tables S2–S4 summarize the results. Similarly to a standard diet, *Wdr72* ^*−/−*^ mice fed with an acid diet exhibited a more alkaline urine. While *Wdr72* ^*−/−*^ males displayed a phenotype comparable to their WT littermates, *Wdr72* ^*−/−*^ females showed a more severe phenotype. Female *Wdr72* ^*−/−*^ mice were unable to increase NH_4_^+^ excretion to the same extent as their WT littermates and developed hyperchloremic metabolic acidosis with lower blood pH and decreased blood HCO_3_^−^. Finally, urea clearance was drastically decreased in *Wdr72* ^*−/−*^ females. However, no difference in the urinary excretion of the kidney injury marker NGAL was observed (Table S2).

### 2.3 WDR72 Protein Abundance in Mouse Kidney Is Altered by Acid–Base Status

**FIGURE 4:**
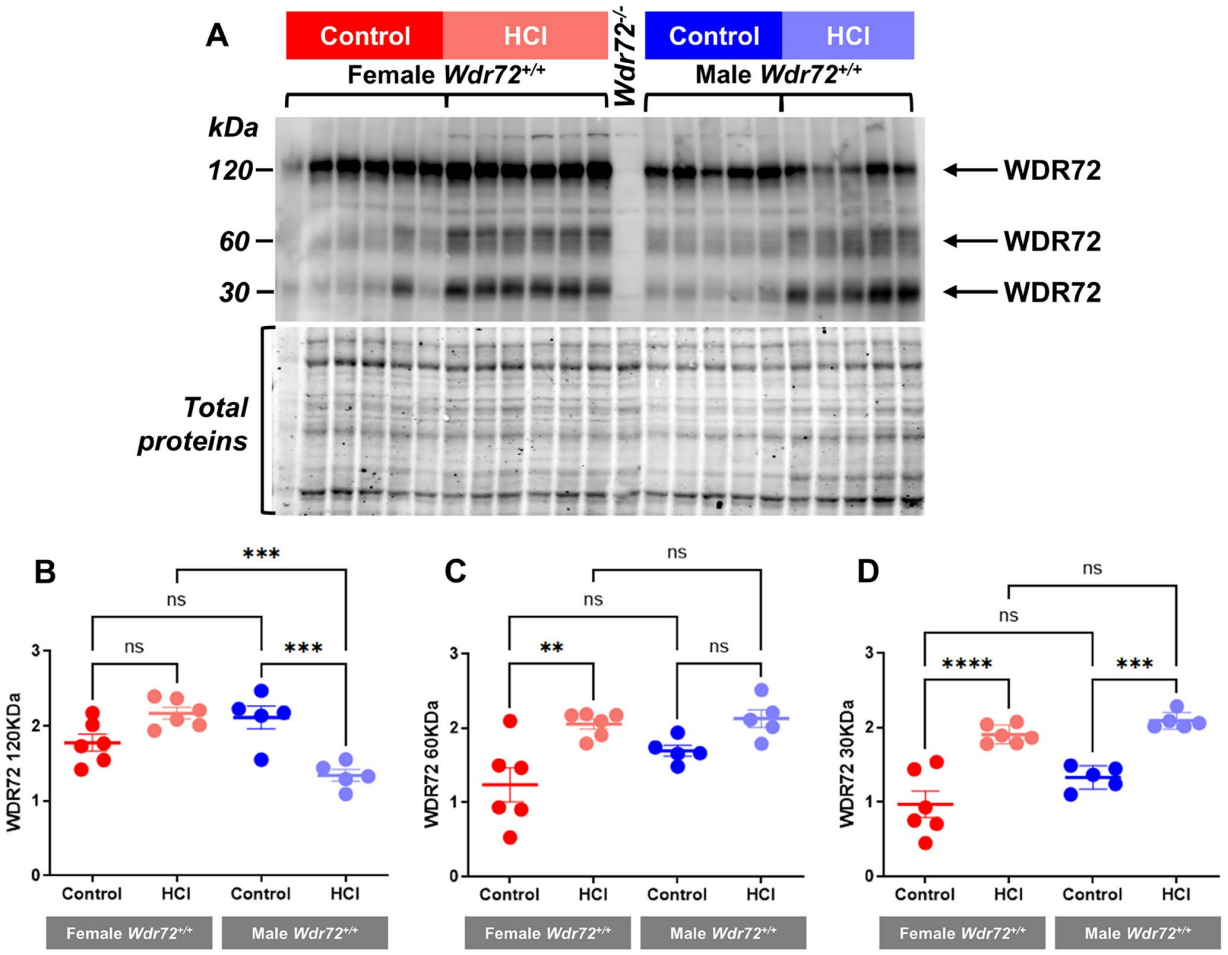
Western blot analysis of WDR72 expression in female and male *Wdr72* ^*+/+*^ mice subjected to either a control or HCl diet. (A) WDR72 protein abundance was evaluated by Western blot analysis of total kidney membrane fractions prepared from female (*n* = 6) and male (*n* = 5) *Wdr72* ^*+/+*^ mice. Membrane fractions from a female *Wdr72* ^*−/−*^ total kidney served as a negative control (lane between female and male samples). (B–D) Bar graphs summarizing the densitometric analysis. Values were normalized to the total protein, presented as mean ± SEM. Statistical significance was determined using one‐way ANOVA. **p ≤ 0.01, ***p ≤ 0.001.

To determine the regulation of WDR72 by acid–base status, we performed western blot analysis on kidney tissues from *Wdr72* ^*+/+*^ female and male mice receiving either standard diet or 4 days of acid loading. Immunoblotting revealed 3 bands at 120 kDa, at 60 kDa, and at 30 kDa in WT kidneys which were absent in kidneys from *Wdr72* ^*−/−*^ mice (Figure 4A and Figure S4). In kidney tissue from acid‐loaded mice, we observed a trend towards an increase in the predicted band at 120 kDa in females and a significant decrease in males. However, the band at 60 kDa was significantly increased in acid‐loaded females but unchanged in acid‐loaded males. This band, never documented before, could be a cleaved form of the 2 β‐propellers predicted as the main structure of the protein by Katsura et al. [3]. Finally, the band at 30 kDa was increased to the same extent in both sexes by the acid diet. These results are consistent with the complex structure of WDR72, which may be regulated by cleavage or other posttranslational processes (Figure 4B–D).

### 2.4 H^+^‐ATPases Subunits Localization Is Preserved in Female *Wdr72* ^*−/−*^ Kidney Tissue, but Their Expression and Function Are Altered

Urinary acidification is achieved by the action of H^+^‐ATPases located in type A intercalated cells [29]. To further understand the role of WDR72 in the activity of type A‐IC, we focused on the regulation of the multimeric protein H^+^‐ATPase and its interactome protein RhCG, two protagonists of type A‐IC function and used 4‐day acid loaded females as acid‐loaded *Wdr72* ^*−/−*^ females exhibited the strongest metabolic phenotype.

**FIGURE 5:**
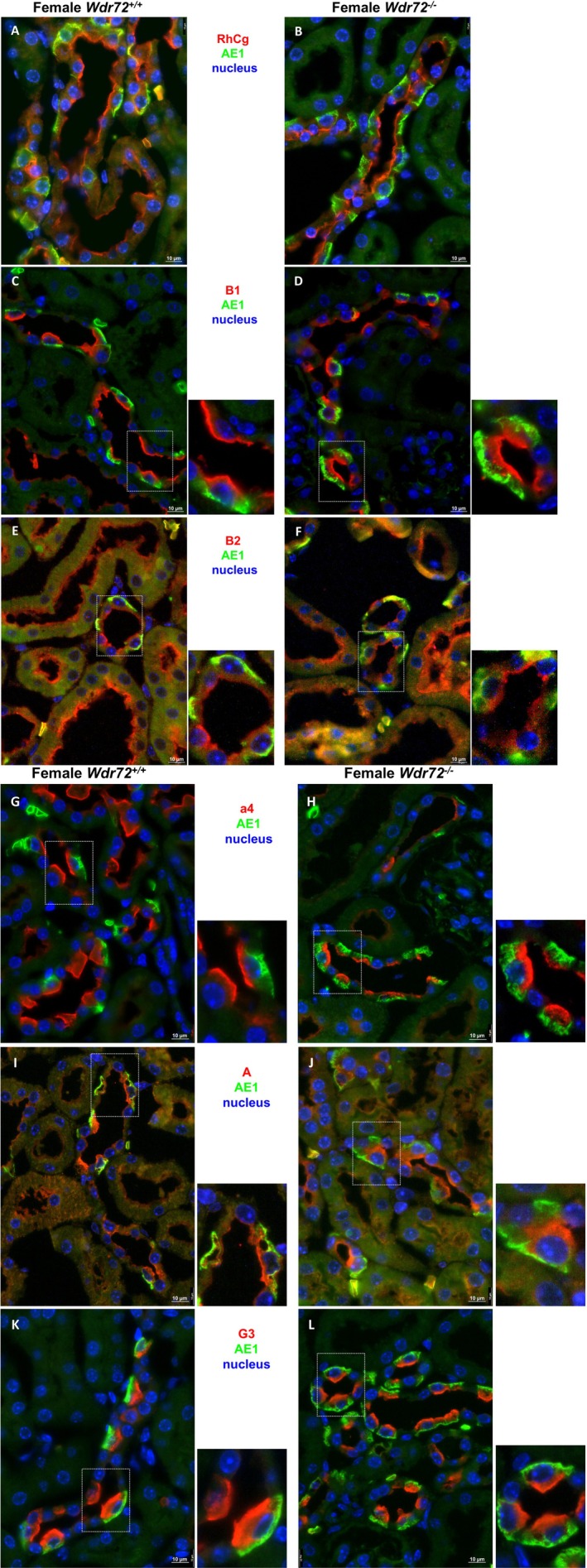
Immunolocalization of H^+^‐ATPases subunits and RhCG at the luminal side of type‐A IC from 4 days HCl treated female mice. Kidney sections from *Wdr72* ^*+/+*^ and *Wdr72* ^*−/−*^ female mice treated with a 4‐day HCl diet were stained to visualize AE1 protein (green), marking type‐A IC, and nuclei in blue using DAPI. Proteins identified (in red) comprise: (A and B) RhCG, (C and D) the H^+^‐ATPase subunits B1, (E and F) B2, (G and H) a4, (I and J) A, and (K and L) G3. All were appropriately localized to the luminal plasma membrane of AE1‐positive cells with no differences observed between genotypes. Original magnification 400×.

We examined the localization of 5 different H^+^‐ATPase subunits, B1, B2, and A—three subunits part of the cytoplasmic V_1_ domain, and a4 and G3, two subunits of the V_0_ domain. We also stained the RhCG ammonia channel, which we have previously shown to be part of the interactome of the multimeric protein H^+^‐ATPase [27]. No differences were observed in the subcellular localization of any of these proteins in the type A‐IC, which were identified through staining for the basolateral Cl/HCO_3_ exchanger AE1 (Figure 5).

**FIGURE 6:**
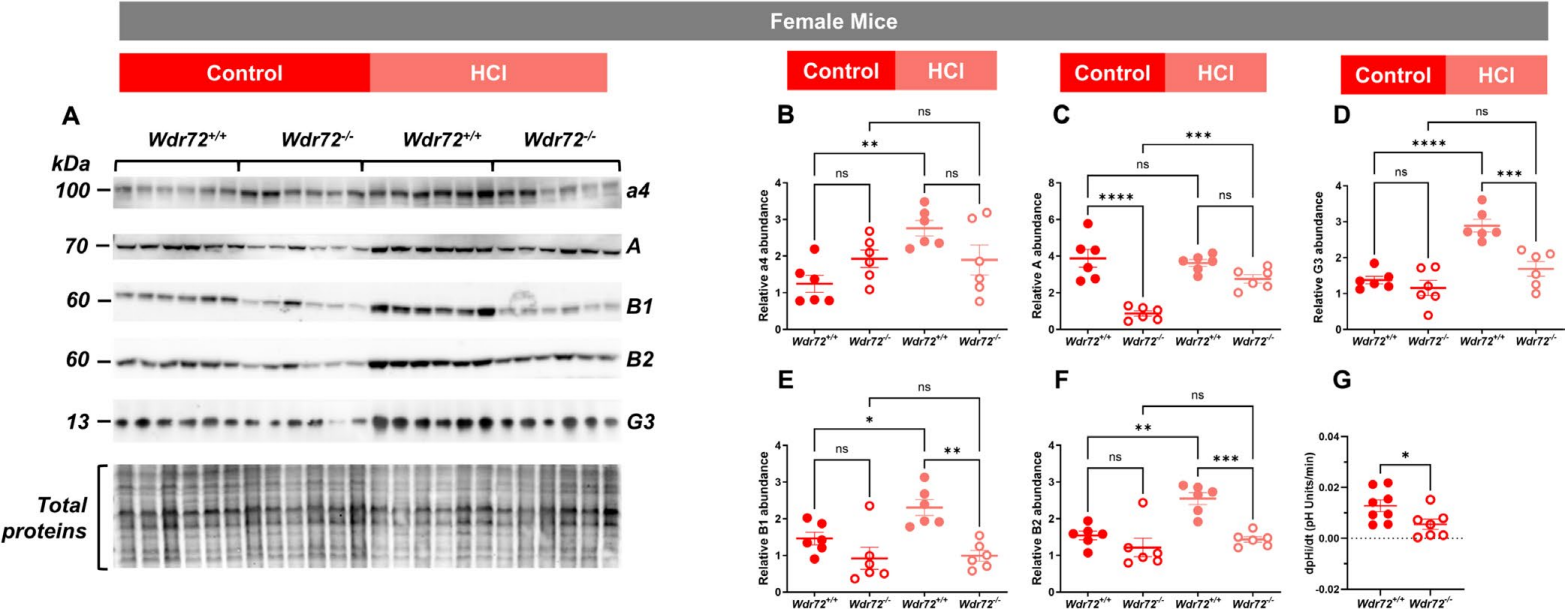
Reduced H^+^‐ATPase subunit expression and activity in *Wdr72* ^*−/−*^ kidneys. (A) The abundance of the a4, A, B1, B2, and G3 H^+^‐ATPase subunits was assessed by Western blot analysis of total kidney membrane fractions from female *Wdr72* ^*+/+*^ and *Wdr72* ^*−/−*^ mice (*n* = 6 per group) maintained on either a control or HCl diet. (B–F) Bar graphs summarizing the densitometric analyses. Values were normalized to total protein content and are presented as mean ± SEM. Statistical significance was evaluated using one‐way ANOVA. **p ≤ 0.01, ***p ≤ 0.001, ****p ≤ 0.0001. (G) Cortical collecting ducts were isolated from the kidneys of *Wdr72* ^+/+^ and *Wdr72* ^*−/−*^ mice, microperfused in vitro, and monitored for intracellular pH (pH_i_). A 20 mM NH_4_Cl solution was applied to the basolateral perfusate. The graph summarizes dpHi/dt values following removal of the luminal NH_4_Cl pulse (*n* = 7–8 tubules per group). Values are as means ± SEM. Statistical significance was assessed by unpaired *t*‐test. *p ≤ 0.05 versus *Wdr72* ^*+/+*^ mice.

Immunoblotting of the five H^+^‐ATPase subunits in membrane fractions from *Wdr72* ^*+/+*^ and *Wdr72* ^*−/−*^ kidneys revealed that, in wild‐type mice, all subunits except subunit A were upregulated following acid challenge. In contrast, this acid‐induced upregulation was completely abolished for subunit a4 and even reversed for B1, B2, and G3, resulting in a downregulation of the subunits in membrane fractions from *Wdr72* ^*−/−*^ kidneys. Notably, expression of subunit A was already markedly reduced in *Wdr72* ^*−/−*^ kidneys under standard diet conditions. Although a slight increase is observed upon acid loading, its level only reached that observed in wild‐type kidneys (Figure 6A–F).

We also performed immunoblot analyses for subunits a4, A, B1, and B2 in membrane fractions from male kidneys. Conversely, in wild‐type males, all four subunits remained unchanged after acid loading. In *Wdr72* ^*−/−*^ males, the a4 subunit was unaffected by diet, while subunits A, B1, and B2 were consistently decreased compared to wild‐type littermates, regardless of dietary conditions (Figure S5). Finally, we measured H^+^‐ATPase activity in isolated microperfused cortical collecting ducts (CCDs) from female mice under a standard diet. H^+^‐ATPase activity was significantly reduced in CCDs from *Wdr72* ^*−/−*^ mice compared to *Wdr72* ^*+/+*^ littermates (Figure 6G, Figure S6).

### 2.5 Compensatory and Deleterious Mechanisms Are Established Along the Kidney Segments in *Wdr72* ^*−/−*^ Mice

We performed further immunoblotting to examine expression of key players of acid–base handling along the nephron in kidney tissue from female *Wdr72* ^*+/+*^ and *Wdr72* ^*−/−*^ mice subjected to either a standard diet or a 4‐day acid load. Under normal dietary conditions, female *Wdr72* ^*−/−*^ displayed an increase in PEPCK‐C expression, an enzyme located in the proximal tubule that contributes to renal ammonium production, as well as a reduction of the Cl^−^/HCO_3_^−^ cotransporter pendrin (PDS), which mediates HCO_3_^−^ secretion by the type B‐IC of the collecting duct. Upon 4 days of acid loading, the Na^+^‐H^+^ exchanger NHE3, responsible for proton secretion and HCO_3_^−^ reabsorption in the proximal tubule and the thick ascending limb, was appropriately upregulated in both *Wdr72* ^*+/+*^ and *Wdr72* ^*−/−*^ kidney tissues. PEPCK‐C and NKCC2, which play crucial roles in ammonia handling by the proximal tubule and thick ascending limb, respectively, increased in *Wdr72* ^*+/+*^ but not in *Wdr72* ^*−/−*^ kidneys. NBCe1, the Na^+^/HCO_3_^−^ cotransporter in the proximal tubule, was markedly reduced to a similar extent in both genotypes. Additionally, PDS expression remained low in *Wdr72* ^*−/−*^ kidneys. Interestingly, the proximal tubule‐specific potassium channel Kir 4.2 was unchanged during acidosis in *Wdr72* ^*+/+*^ but was upregulated in *Wdr72* ^*−/−*^ kidneys (Figure S7A–G).

### 2.6 No Defect in Calcium Handling Was Observed in *Wdr72* ^*−/−*^ Mice

Given the reported link between WDR72 and uromodulin excretion [12], we examined its urinary excretion and found no difference between genotypes (Figure S8A). Moreover, *WDR72* has been linked to nephrocalcinosis and kidney stones [6]. We found no significant differences in parameters such as serum calcium levels or urinary calcium excretion (Figure S8B,C). To detect renal calcium deposits, we performed Alizarin Red staining. While prominent calcium deposition was evident in *Clcn10* ^*−/−*^ kidney tissue, a model with demonstrated nephrocalcinosis [30], no staining was observed in *Wdr72* ^*−/−*^ kidney tissue (Figure S8D).

## 3 Discussion

Patients with amelogenesis imperfecta and distal renal tubular acidosis carry biallelic genetic variants in *WDR72*. Our study provides a mechanistic and molecular explanation for a role of WDR72 in urinary acidification and reveals an essential contribution to acid–base homeostasis. We demonstrate that *Wdr72* mRNA is ubiquitously expressed along the nephron, while WDR72 protein was specifically detected in type A‐IC in the collecting duct. Moreover, our functional analyses reveal that *Wdr72* deletion disrupts urinary acidification, particularly in female mice, leading to a severe metabolic acidosis phenotype. Furthermore, our findings suggest a complex regulation of WDR72 protein abundance by acid–base status and an intricate interplay between *Wdr72* expression and H^+^‐ATPase subunits' abundance and H^+^‐ATPase activity.

### 3.1 WDR72 Expression in the Kidney

WDR72 expression is restricted to few tissues including kidney. Here, we demonstrate that *Wdr72* mRNA is found throughout the entire tubular system, including the segments of the proximal tubule, thick ascending limbs, and collecting ducts, with no significant sex differences. Similarly, in healthy human kidney, the broad mRNA expression pattern suggests a potential role for *WDR72* along the nephron. However, in WT mouse tissue, WDR72 protein was only detectable at the apical membrane of type A and B intercalated cells. Our studies here focused on the role of WDR72 in type A intercalated cells to elucidate the role of this protein in patients with dRTA and biallelic variants in the *WDR72* gene. However, the prominent expression of *WDR72* in type B intercalated cells warrants further investigations. It remains unclear whether low levels of protein could not be detected with the antibodies available or whether this represents evidence for posttranscriptional mechanisms controlling WDR72 protein expression, potentially through cell‐type‐specific translation, trafficking, or degradation. The absence of detectable WDR72 protein in other nephron segments despite its presence on the mRNA level may indicate that its main functional role in young adult animals may be restricted to intercalated cells. However, its role in other cell types may be more subtle or require additional factors such as aging, cellular stress, or kidney disease to be uncovered. This expanded function is supported by multiple GWAS studies linking *WDR72* to kidney function and disease in humans [13, 15, 31, 32, 33, 34, 35].

### 3.2 Regulation of WDR72 Protein by Acid–Base Status

Western blot analysis of *Wdr72* ^*+/+*^ mouse whole kidney membrane fractions showed three WDR72 bands, absent in *Wdr72* ^*−/−*^ tissue, confirming specificity. The predicted 120 kDa band was differentially regulated by acid loading. The increased expression in females and decrease in males indicates a sex‐specific posttranscriptional regulation. This supports the idea of sex‐specific regulatory mechanisms in kidney epithelial cells, similar to those observed by Harris et al. for proteins involved in renal ammonia metabolism [36, 37, 38]. In this context, it is plausible that the WDR72 homolog, WDR7, also expressed in type‐A IC, may partly take over the role of WDR72 in male kidneys [18, 22]. However, we were not able to detect WDR7 protein expression in our samples and its mRNA expression remains low compared to *Wdr72* in both humans and mouse kidneys as observed in single‐cell RNA‐sequencing data sets (Figure 2A–C [24, 39]). Additionally, upon acid loading, a 60 kDa band, potentially representing a cleaved WDR72 fragment, was markedly upregulated in female kidneys, whereas the 30 kDa band was upregulated in both sexes. To our knowledge, this is the first observation of potential WDR72 cleavage in epithelial cells in response to acid–base disturbances. Whether the observed regulation of WDR72 abundance reflects only changes at the level of type A intercalated cells or also other cell types remains to be established. Collectively, these results provide novel evidence that WDR72 expression and function are tightly regulated by acid–base balance, reinforcing its role in acid–base homeostasis.

### 3.3 Sex‐Specific Metabolic Acidosis and Renal Adaptation in *Wdr72* ^*−/−*^ Mice

Only *Wdr72* ^*−/−*^ females developed a severe incomplete dRTA upon acid loading. Sex differences in acid handling have previously been demonstrated in mice [36, 37]. These studies uncovered sex‐specific differences in kidney structure, showing that males possess larger proximal tubules, while females display a greater volume of collecting ducts [36, 37]. These structural variations may suggest a more prominent role of the collecting duct in acid–base homeostasis in females, potentially contributing to the more severe phenotype observed in *Wdr72* ^*−/−*^ females. In our study, we observed sex‐specific differences in the regulation of H^+^‐ATPase subunit expression across genotypes and dietary conditions. Notably, females relied more than males on the adaptive upregulation of most subunits (except A) in response to acid load, a response that is abolished or reversed in *Wdr72* ^*−/−*^ mice. Conversely, *Wdr72* deficiency in males primarily affects basal subunit expression, with minimal impact on acid‐inducible changes.

Of note, our study was not primarily designed to study sex differences, and we did not directly compare expression of acid–base transporters between sexes. Moreover, it appears that females experienced a higher effective acid load at Day 4, which may contribute to the differences observed.

Sex differences have not yet been addressed in human carriers of *WDR72* variants. However, a recent report [40] investigated the genetic spectrum and long‐term outcomes of 56 dRTA patients from a large Indian cohort, highlighting the prominent role of *WDR72* in the diagnosis of dRTA. In that study, causative *WDR72* variants were identified in 14% of cases, with a striking female predominance (~89%) compared with 30% females in the overall cohort. However, a study specifically designed to explore the link between *WDR72* variants and sex differences in dRTA remains necessary to clarify this observation. We also observed compensatory acid–base handling by more proximal segments, including the proximal tubule and thick ascending limb. Notably, the basolateral K^+^ channel KIR4.2 in the proximal tubule was upregulated in *Wdr72* ^*−/−*^ kidneys during acidosis, potentially representing an adaptive response to compensate for impaired distal acid handling. This is consistent with the known role of KIR4.2 in regulating ammoniagenesis and ammonia transport in the proximal tubule [41]. Finally, the observation that female *Wdr72* ^*−/−*^ mice exhibited reduced urea clearance with an increase in blood urea levels, which is usually considered as an early marker of kidney injury [42], suggests a functional impairment of kidneys which could lead to chronic kidney damage [42].

### 3.4 *Wdr72* Deletion Impairs H^+^‐ATPase Function in Type A‐IC

Because inactivating *WDR72* variants lead to distal renal tubular acidosis in humans [4, 6, 7, 8] and due to the prominent localization of WDR72 in intercalated cells in our study, we investigated its role in the regulation of H^+^‐ATPase activity. H^+^‐ATPases are crucial for IC function and are primarily active when localized at the plasma membrane [21, 43]. Our data demonstrate that the subcellular localization of H^+^‐ATPase subunits shows no major abnormality in *Wdr72* ^*−/−*^ type A‐ICs, suggesting WDR72 is not essential for subunit trafficking. However, western blot analysis on total kidney membrane fractions indicated a dysregulation of the abundance of H^+^‐ATPase subunits, which was paralleled by reduced H^+^‐ATPase activity in isolated perfused cortical collecting duct. Dysregulation at the level of the whole kidney, on all tested subunits, is not common in the case of a defect solely in IC. Our recent study on *Atp6v1b1* ^*−/−*^ mice serves as a strong example, as we observed impaired assembly of the H^+^‐ATPase in ICs due to the absence of the B1 subunit [44]. However, at the whole kidney tissue level, we had not detected any abnormalities in the expression of the other subunits examined [44]. This suggest that the role of WDR72 in the regulation of H^+^‐ATPase subunits may not be restricted to the collecting duct but potentially includes other nephron segments. The way WDR proteins regulate H^+^‐ATPase activity in the kidney is not well understood to date. WDR72 may be part of the Rabconnectin complex, which interacts with and regulates H^+^‐ATPase assembly, as observed in yeast where RAVE, the yeast Rabconnectin complex equivalent, is a known regulator of H^+^‐ATPase holoenzyme assembly [18, 45]. In the brain, the two rabconnectin protagonists, the rabconnectin‐β, WDR7 and the Rabconnectin‐α, DMXL2, members of the eukaryotic Rabconnectin complex, interact closely [46, 47] and have been implicated in the regulation of Notch signaling as well as the assembly and activity of vesicular H^+^‐ATPase [48, 49]. In the kidney, WDR7 and the DMXL1 isoforms are part of the H^+^‐ATPase interactome [22]. Notably, WDR7 shares about 40% sequence homology with WDR72, suggesting functional similarities [18, 50]. Additionally, DMXL1 knockout mice have an impaired acid handling due to a disrupted assembly of the *V*_0_ and *V*_1_ domains of the H^+^‐ATPase holoenzyme [17] In this study, we demonstrate that *DMXL1* and *WDR72* mRNA share the same expression pattern along the nephron in both humans and mice. Since WDR72 may, like DMXL1, be part of Rabconnectin to form a complete Rabconnectin complex, a known regulator of H^+^‐ATPase assembly and function, its absence could impair subunit stability, assembly, or coordinated regulation, potentially leading to defective proton excretion and acid–base imbalance [18].

### 3.5 Conclusion

Our study reveals a key role for WDR72 in renal acid–base homeostasis through its regulation of H^+^‐ATPase expression and function in intercalated cells and explains the development of dRTA in patients with biallelic loss‐of‐function variants in this gene. Despite widespread mRNA expression, WDR72 protein is mostly expressed in intercalated cells in mouse kidney. *Wdr72* deletion reduces urinary acid secretion and leads to severe, sex‐dependent metabolic acidosis in female mice. Finally, the extensive nephron‐wide distribution of *WDR72* implies further, yet undefined, contributions to kidney function.

## 4 Methods

### 4.1 Animals

The mouse strain used for this research project, C57BL/6J‐*Wdr72* ^*tm1.1Jpsi*^/Mmucd, RRID:MMRRC_037475‐UCD, was obtained from the Mutant Mouse Resource and Research Center (MMRRC) at University of California at Davis, an NIH‐funded strain repository, and was donated to the MMRRC by James Simmer, Ph.D., University of Michigan, Jan Ching Chun Hu, Ph.D., University of Michigan. *Wdr72* Wild‐type and knock‐out mice (*Wdr72* ^*+/+*^ and *Wdr7*2^−/−^, respectively) were genotyped as previously described [51].

All experiments were performed according to Swiss Animal Welfare laws and were approved by the local veterinary authority (Veterinäramt Zürich) under the license numbers ZH134/2020 and ZH013/2024. *Wdr72* mice used in this study were in a range of ages from 60 to 100 days old. Only littermates were used for experiments.

Mice were housed in metabolic cages (Techniplast, Switzerland) for 5 days. Mice were fed with a standard powdered laboratory chow (Kliba, Augst, Switzerland) and given distilled water ad libitum to adapt to metabolic cages for 1 day. Mice were then given an HCl‐containing diet (50:75 w/v 0.33 M HCl added to powdered standard chow) for 4 days. The daily food and water intake, urine output, and body weight were monitored throughout the study. 24‐h urine was collected daily under light mineral oil in the urine collector to determine urinary parameters. On Day 5, retro‐orbital blood samples were collected under light anesthesia with isoflurane to assess blood gas status. Animals were sacrificed via exsanguination under isoflurane anesthesia. Blood samples were collected, and plasma was immediately isolated via centrifugation. Kidneys were harvested after euthanasia.

### 4.2 Analytic Procedures

Blood pH, pCO_2_, HCO_3_^−^, and electrolytes were measured with a blood gas analyzer (Epoc blood gas analysis system, Siemens, Germany). Urinary pH was measured on freshly collected 24‐h urine with a pH meter (Metrohm AG, Zofingen, Switzerland). Urine and blood creatinine, urine phosphate, ammonium, and calcium were measured on a UniCel DxC 800 Synchron Clinical System Automat (Beckman Coulter GmbH, Germany).

### 4.3 Uromodulin Elisa

The mouse Uromodulin ELISA Kit (single‐wash, 90‐min SimpleStep ELISA, ab245726 (ABCAM, Cambridge, UK)) was used to quantitatively measure Uromodulin in mouse urine samples, following the manufacturer's recommendations.

### 4.4 Immunohistochemistry

#### 4.4.1 Organ Fixation

*Wdr72* ^*+/+*^ and *Wdr72* ^*−/−*^ mice either control diet or four‐day HCl‐loaded were anesthetized with isoflurane and perfused through the left heart ventricle with a prefixative solution (1000 U/mL heparin, 0.2% procaine–HCl, 3.2% CaCl_2_, and 0.18% NaCl) followed by the fixative 3% paraformaldehyde in PBS. After incubation in 4% paraformaldehyde/PBS for 1 h, kidneys were placed overnight in 32% sucrose/PBS and subsequently embedded in optimal cutting temperature Embedding Matrix (Cell Path, Newtown, Wales, the United Kingdom) and flash frozen in liquid propane. Cryosections of 1 to 2 μm were mounted on slides (Superfrost Plus, Thermo Scientific).

#### 4.4.2 Alizarin Staining

Kidney sections were rinsed with distilled water and incubated for 2 min in a 20 g/L alizarin red solution (pH 4.2, diluted in distilled water and filtered). They were then rinsed with distilled water for 5 min before undergoing hematoxylin staining for a maximum of 3 min, followed by another rinse in distilled water. Postfixation was carried out sequentially in a 75% ethanol bath, two 100% ethanol baths, and a final xylene bath, each for 5 min. Coverslips were mounted using Glycergel (DakoCytomation, Baar, Switzerland). The sections were then examined using a Leica DMR microscope equipped with a Leica DFC320 camera (Leica Microsystems, Germany).

#### 4.4.3 Immunostaining

Autofluorescence from free aldehyde sites was quenched by incubating slides in a 50 mM NH_4_Cl/PBS solution for 20 min. Sections were then treated with 1% SDS/PBS for 5 min, washed with PBS, and blocked with 1% bovine serum albumin/PBS. After blocking, sections were incubated with primary antibodies (Table S5) diluted in 1% BSA in PBS for incubation overnight at 4°C. Then, samples were washed 3 times with PBS and incubated with the secondary antibodies (donkey anti‐rabbit 594, donkey anti‐guinea pig 488 and donkey anti‐goat pig 647) (Thermofisher Scientific, Dreieich, Germany) at 1:1000 and DAPI (4′,6‐diamidino‐2‐phenylindole) for 2 h at room temperature. After three consecutive washing steps with PBS, coverslips were mounted with Glycergel (DakoCytomation, Baar, Switzerland). Sections were visualized with a Leica DMR microscope equipped with a Leica DFC320 camera (Leica microsystems, Germany). Images were transferred by a Leica TFC Twain 6.1.0 program and processed using Adobe Photoshop.

#### 4.4.4 RNAscope

The RNAscope Singleplex Fluorescent Assay kit was used as per manufacturer's instructions (RNAscope 2.5 HD Assay—RED, Advanced Cell Diagnostics Inc. Newark, CA) on 4% PFA fixed tissue sections (1–2 μm) from four‐day HCl‐loaded *Wdr72* ^*+/+*^ and *Wdr72* ^*−/−*^ mouse kidneys. Sections were hybridized with probe‐sets targeting *Wdr72* mRNA and counterstained with 4′,6‐diamidino‐2‐phenylindole (DAPI). Positive and negative probes on *Wdr72* ^*+/+*^ kidney tissues as well as *Wdr72* ^*−/−*^ kidney tissues were used as controls (Figures S1 and S2). Co‐detection of *Wdr72* mRNA with anti‐NaPiIIa, anti‐NKCC2 and anti‐AE1 antibodies (Table S5) followed by secondary antibody (Goat Anti‐Rabbit IgG Alexa Fluor488; ab150081,1:1000) was performed according to manufacturer's instructions. Sections were visualized with a Leica DMR microscope equipped with a Leica DFC320 camera (Leica microsystems, Germany) in Bright‐field microscopy and fluorescence microscopy. Images were transferred by a Leica TFC Twain 6.1.0 program and processed using Adobe Photoshop.

### 4.5 Immunoblotting

Total membrane fractions were prepared from *Wdr72* ^*+/+*^ and *Wdr72* ^*−/−*^ mouse kidneys. Frozen kidneys were homogenized in an ice‐cold K‐HEPES buffer (200 mM mannitol, 80 mM HEPES, 41 mM KOH, pH 7.5) containing a protease inhibitor mix (Complete Mini, Roche Diagnostics, Germany) at a final concentration of 1 tablet in a volume of 10 mL solution. The samples were centrifuged at 1000 g for 20 min at 4°C. Subsequently, the supernatant was transferred to a new tube and centrifuged at 161000 g for 30 min at 4°C. The pellet was resuspended in K‐HEPES buffer containing protease inhibitors.

Up to fifty micrograms of total membrane proteins were solubilized in Laemmli loading buffer containing 10% DTT and separated into 8 to 10% polyacrylamide gels. For immunoblotting, proteins were transferred by electrophoresis to polyvinylidene fluoride membranes (Immobilon‐P, Millipore Corp., Bedford, MA, USA). Total protein was quantified using the Revert Total Protein Stains for Western Blot Normalization assay and an Odyssey Imaging system following the manufacturers' protocol (LI‐COR Biosciences, NE USA). After blocking with 5% milk powder in PBS for 60 min, blots were incubated with primary antibodies (see Table S5) overnight at 4°C. After washing in PBS/0.01% Tween, membranes were incubated for 2 h at room temperature with secondary goat anti‐rabbit or donkey anti‐mouse antibodies 1:5000 linked to alkaline phosphatase (Promega, Madison, WI, USA). The protein signal was detected with the substrate chemiluminescent CDP‐*Star* (Sigma Aldrich, USA) using the las‐4000 image analyzer system (Fujifilm Life Science USA). All images were analyzed using ImageJ to calculate the protein of interest total protein ratio.

### 4.6 Microperfusion Studies

The composition of the various solutions is given in Table S6. In nominally Na^+^‐free solutions, Na^+^ was replaced isosmotically with N‐methyl‐D‐Glucamine (NMDG^+^). Cl^−^‐free solutions contained equimolar gluconate as a replacement for Cl^−^. We compensated for Ca^2+^ chelated by Cl^−^ substitutes by increasing total Ca^2+^ concentration from 2 to 7.5 mM in Cl^−^‐free solutions.

#### 4.6.1 Tubule Isolation

For the isolation of tubules, female mice were deeply anesthetized with Xylazin/Ketamin i.p injection. Both kidneys were removed and cut into thin coronal slices for tubule dissection. Cortical collecting ducts (CCDs) were isolated from corticomedullary rays at 6°C in the dissection solution (Table S6 solution A) under a dissecting microscope with sharpened forceps.

#### 4.6.2 Intracellular pH Measurement in Isolated CCDs

Microperfusion experiments were conducted as previously described [27]. Briefly, dissected CCDs were transferred to the bath chamber on the stage of an inverted spinning disc microscope (IX81, Olympus, Japon). The average tubule length exposed to bath fluid was limited to 300 μm to prevent motion of the tubule. Intracellular pH in CCD cells was assessed with imaging‐based, dual excitation‐wavelength fluorescence microscopy with use of the fluorescent probe 2′,7′‐Bis‐(2‐Carboxyethyl)‐5‐(and‐6)‐Carboxyfluorescein, Acetoxymethyl Ester (BCECF‐AM, Invitrogen). Tubules were loaded for 20 min at 37°C with 5 μM of the BCECF‐AM added to the peritubular fluid. The loading solution was then washed out to remove all non‐de‐esterified dye by initiation of bath flow and the tubule was equilibrated with a dye‐free bath solution for 10 min. Bath solution was warmed to 37°C by water jacket immediately upstream to the chamber.

Intracellular dye was excited alternatively every 2 s at 440 and 505 nm with a LedHub, (Omicron, Germany). Emitted light was collected through a dichroic mirror, passed through a 530 nm filter, and focused onto an OrcaFusion BT camera (Hammamatsu, Japon) connected to a computer. The measured light intensities were digitalized with the Cell^M^&Cell^R^ Imaging hardware system (Evident, Japan) for further analysis. For each tubule, 1 to 4 non‐type‐A IC were analyzed, and the mean gray level was measured. Intracellular dye was calibrated at the end of each experiment using the high [K^+^]‐nigericin technique. Tubules were perfused and bathed with a HEPES‐buffered, 90‐mM K^+^ solution (Table S6 solution B) containing 10 μM of the K^+^/ H^+^ exchanger nigericin (Sigma Aldrich, Buchs, Switzerland). Four different calibration solutions, titrated with Tris to pH 5, 6, 7, and 8 were used.

#### 4.6.3 H^+^‐ATPase Activity Measurements in Cortical Collecting Ducts

After loading with BCECF‐AM, intercalated cells were identified, and fluorescence recording was initiated for 2 min and the bath solution was changed to a solution containing 20 mM NH_4_Cl (Table S6 solution D) for 2 min that elicited a rapid intracellular alkalinization, followed by a sharp acidification. After the acidification reached a plateau, NH_4_Cl was removed from the bath (Table S6 solution C) to further acidify cells and initiate direct H^+^‐excretion with a maximum rate (initial steep intracellular alkalinization) to determine H^+^‐ATPase activity as previously demonstrated [27, 44].

### 4.7 Single Cell RNA‐Seq Analysis

Previously published human (GSE151302) [23] single nuclei and mouse single‐cell (GSE129798) [24]. RNA‐seq datasets were downloaded and processed using Seurat 5.3.0. The human dataset was analyzed using only healthy controls, following the instructions of the Github code (https://github.com/p4rkerw/Muto_Wilson_NComm_2020/blob/master/snRNA_prep/seurat_rna_process.R). The mouse dataset was stratified by sex category and analyzed independently. We used a preprocessed dataset that was filtered for genes/cells (1,000–4,000), mRNA transcripts/cell (1,000–16,000) and mitochondrial genes/cells (< 35%). Normalization, feature selection for highly variable features and data scaling was performed using the *NormalizeData*, *FindVariableGenes* and *ScaleData* functions, respectively. Linear dimensionality reduction with the *RunPCA* function identified 30 useful principal components, used for clustering with the *FindNeighbours* and *FindClusters* functions (resolution of 0.5) and for nonlinear dimensionality reduction with the *RunUMAP* function. The ontology of the original dataset was used for annotation. Data were visualized with the *DotPlot* function.

### 4.8 Statistics

GraphPad Prism 10 was used for data analysis and representation. Data are shown as means ± SEM. Comparisons between experimental conditions were performed using nonparametric ANOVA (Kruskal–Wallis test) or one‐way or two‐way ANOVA of variance with Bonferroni test. *p* values of ≤ 0.05 were considered statistically significant.

## Author Contributions

**Hannah Auwerx:** performed experiments, analyzed data. **Moana Busch‐Dohr:** performed experiments. **Xiaoxu Li:** analyzed data. **Carsten A. Wagner:** funding acquisition, writing – review and editing, writing – original draft, supervision, resources, project administration, conceptualization. **Soline Bourgeois:** funding acquisition, conceptualization, performed experiments, analyzed data, writing – original draft, supervision. All authors read, edited, and approved the manuscript.

## Funding

This study was supported by a grant from the Swiss Nationals Science Foundation (212303) to Carsten A. Wagner and a grant from the Swiss Life Jubiläumsstiftung to Soline Bourgeois.

## Ethics Statement

All animal experiments were approved by the local veterinary authority (Veterinäramt Zürich) under the license numbers (ZH134/2020 and ZH013/2024).

## Conflicts of Interest

Carsten A. Wagner reports honoraria from Kyowa Kirin and collaborations with Bayer AG and NovoNordisk outside this manuscript.

## Supporting information


**Figure S1:** Bright‐field visualization of *Wdr72* mRNA expression in mouse renal tubules, including positive and negative controls. Kidney sections from *Wdr72* ^*+/+*^ and *Wdr72* ^*−/−*^ mice treated with a 4 days HCl diet. Pictures were acquired on bright‐field microscopy and signal detected as red dots. (A–C) *Wdr72* probe on (A and B) *Wdr72* ^*+/+*^ and (C) *Wdr72* ^*−/−*^ kidney tissue, (D) negative control probe on *Wdr72* ^*+/+*^ kidney tissue, and (E) positive control probe on *Wdr72* ^*−/−*^ kidney tissue. Original magnification 400×.
**Figure S2:** Positive and negative controls of Wdr72 mRNA detection. Kidney sections from Wdr72+/+ and Wdr72−/− mice treated with a 4 days HCl diet, were co‐stained with antibodies against; (A, D and G) NaPiIIa, (B, E and H) NKCC2 or (C, F and I) (all in green) together with the (A–C) Wdr72 probe, (D–F) a positive control probe, or (G–I) a negative control probe. Nuclei were marked in blue using DAPI. (A–C) No Wdr72‐related staining was detected in kidney tissue from Wdr72−/− mice. Original magnification 400×.
**Figure S3:** Immunolocalization of Wdr72 at the apical side of type B intercalated cells from male C57Bl6 wild‐type mouse subjected to 4 days of alkali loading with DOCA/HCO_3_^−^. Mice received deoxycorticosterone (DOCA)/NaHCO₃ treatment via drinking water containing 0.28 M NaHCO₃ and 2% sucrose, along with a single intraperitoneal injection of 2 mg DOCA dissolved in 50 μL DMSO at the start of the 4‐day treatment period. Kidney sections were co‐stained with antibodies against pendrin (PDS, green) and WDR72 (red) together with DAPI in blue. WDR72 was expressed principally at the luminal side of PDS positive cells. Original magnification 400×.
**Figure S4:** Immunoblotting of kidneys from female and male Wdr72+/+ and Wdr72−/− mice. Upper panel: total membrane fractions from female (F) and male (M) Wdr72+/+ and Wdr72−/− mice were tested with an antibody against Wdr72. Three major bands around 120 kDa, 60 kDA, and 30 kDa were detected in kidneys from Wdr72+/+ mice but not from Wdr72−/− mice. Lower panel: the membrane was stained for total protein to assess similar protein loading in all lanes.
**Figure S5:** Western blot analysis of H+‐ATPase subunits expression in kidneys from male Wdr72 mice subjected to either a control or HCl diet. (A) The abundance of the a4, A, B1, and B2 H+‐ATPase subunits was assessed by Western blot analysis of total kidney membrane fractions from males (*n* = 6 per group) maintained on either a control or HCl diet. (B–E) Bar graphs summarizing the densitometric analyses. Values were normalized to total protein content and are presented as mean ± SEM. Statistical significance was evaluated using one‐way ANOVA. **p ≤ 0.01, ***p ≤ 0.001.
**Figure S6:** Parameters assessed in the ex vivo microperfused CCD from Wdr72 mice (A) Representative pHi trace from a CCD exposed to a basolateral NH4Cl pulse. The arrow indicates the rate of intracellular pH (pHi) change measured. The NH4Cl pulse induced a marked intracellular alkalinization/acidification pick, followed by an alkalinization phase (recovery phase) reflecting pHi recovery due to H+ secretion. The initial slope of the recovery phase (dpHi/dt) was measured and significatively reduced in CCD from Wdr72−/− mice. (B–D) Initial, final, and delta pHi values were not different between phenotypes.
**Figure S7:** Western blot analysis of key proteins involved in acid–base handling along the nephron. (A) The abundance of NBCe1, PEPCK, Kir4.2, NHE3, NKCC2, and pendrin (PDS), was assessed by Western blot analysis of total kidney membrane fractions from female Wdr72+/+ and Wdr72−/− mice (*n* = 6 per group) maintained on either a control or HCl diet. (B–G) Bar graphs summarizing the densitometric analyses. Values were normalized to total protein content and are presented as mean ± SEM. Statistical significance was evaluated using one‐way ANOVA. **p ≤ 0.01, ****p ≤ 0.0001.
**Figure S8:** Normal renal uromodulin excretion and calcium handling in Wdr72−/− mice. Following 4 days of HCl treatment, female mice (red dots: filled for Wdr72+/+ and empty for Wdr72−/−) and male Wdr72−/− mice (blue dots) showed (A) normal blood calcium levels, (B) a comparable increase in urinary calcium excretion in response to the acid load, and (C) no difference in uromodulin excretion compared to their Wdr72+/+ littermates. (D) Alizarin staining of kidney sections from Wdr72+/+ and Wdr72−/− mice subjected to a 4‐day HCl diet revealed no calcium deposition in Wdr72 mice. In contrast, positive control tissue from Claudin‐10 (Cldn10) deficient mice displayed calcium deposits, predominantly in the medulla (red staining).

**Table S1:** Blood parameters from female and male *Wdr72* mice subjected to a control diet. Mean ± SEM, *p ≤ 0.05, **p ≤ 0.01, ***p ≤ 0.00 1 versus *Wdr72* ^+/+^ sex‐matched mice. Mean ± SEM, ^#^ *p* ≤ 0.05, ^##^ *p* ≤ 0.01, ^###^ *p* ≤ 0.001, ^####^ *p* ≤ 0.0001 versus same genotype at baseline. In brackets, number of animals. Crea, creatinine; iCa, ionized calcium. In brackets: number of animals.
**Table S2:** Metabolic and urine parameters from control, 2 days and 4 days HCl treated female Wdr72 mice. Values are mean ± SEM, *p < 0.05 versus Wdr72+/+ mice on same diet, ^#^ *p* < 0.05 versus same genotype at baseline, BW, body weight; crea, creatinine. In brackets: number of animals.
**Table S3:** Metabolic and urine parameters from control, 2 days and 4 days HCl treated male Wdr72 mice. Values are mean ± SEM, *p < 0.05 versus Wdr72+/+ mice on same diet, *p* < 0.05 versus same genotype at baseline, BW, body weight; crea, creatinine. In brackets: number of animals.
**Table S4:** Blood parameters from female and male Wdr72+/+ and Wdr72−/− mice subjected to a HCl diet. Mean ± SEM, *p ≤ 0.05, **p ≤ 0.01, ***p ≤ 0.00 1 versus Wdr72+/+ sex‐matched mice. Mean ± SEM, ^#^ *p* ≤ 0.05, ^##^ *p* ≤ 0.01, ^###^ *p* ≤ 0.001, ^####^ *p* ≤ 0.0001 versus same genotype at baseline. In brackets, number of animals. iCa, ionized calcium; crea, creatinine. In brackets: number of animals.
**Table S5:** Primary antibodies used in this study.
**Table S6:** Composition of microperfusion solutions.

## Data Availability

All data are included in this manuscript. Original data can be made available upon reasonable request.
