# Supplementary material for: WDR72 Is Required for Urinary Acidification and Normal H+‐ATPase Activity in Intercalated Cells in Mice
Source: Acta Physiol (Oxf). 2026 Feb 4;242(3):e70165. doi: 10.1111/apha.70165 (PMC12869471; doi:10.1111/apha.70165)

Suppl. Figure 1 **Auwerx et al.**

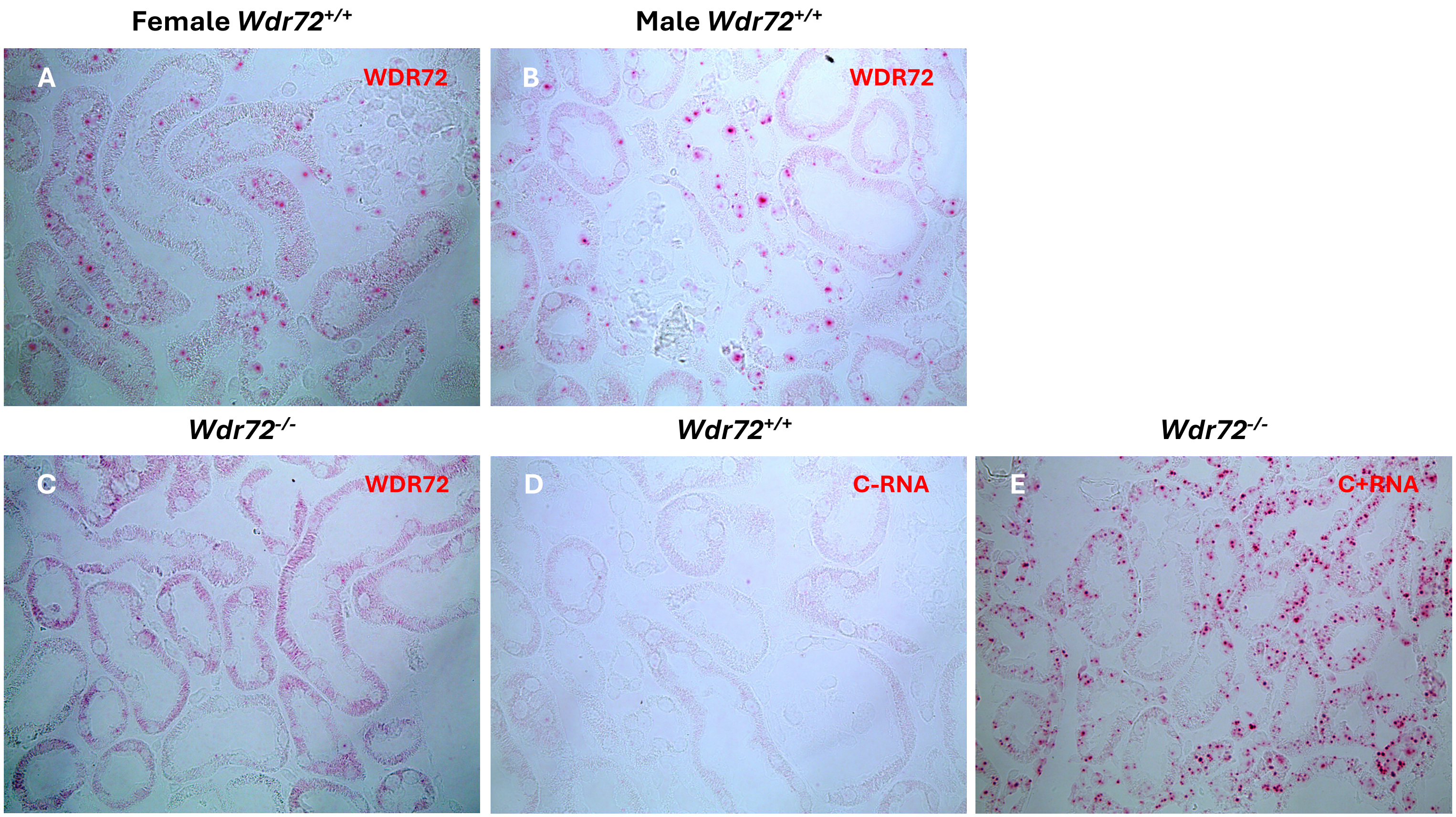

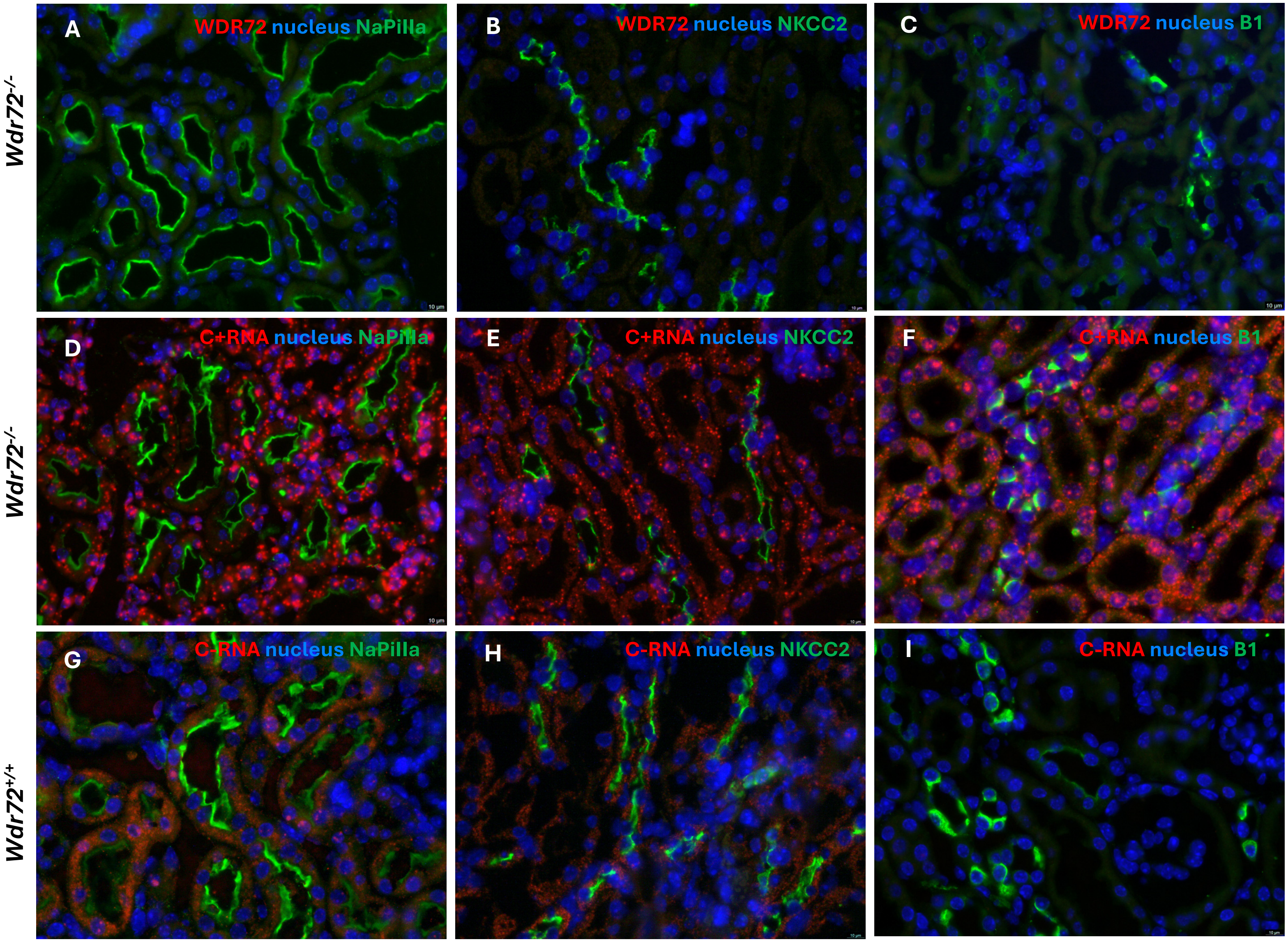

Suppl. Figure 2
Auwerx et al.

Male C57Bl6 wild-type mouse subjected to 4 days of alkali loading with DOCA/HCO_3^-

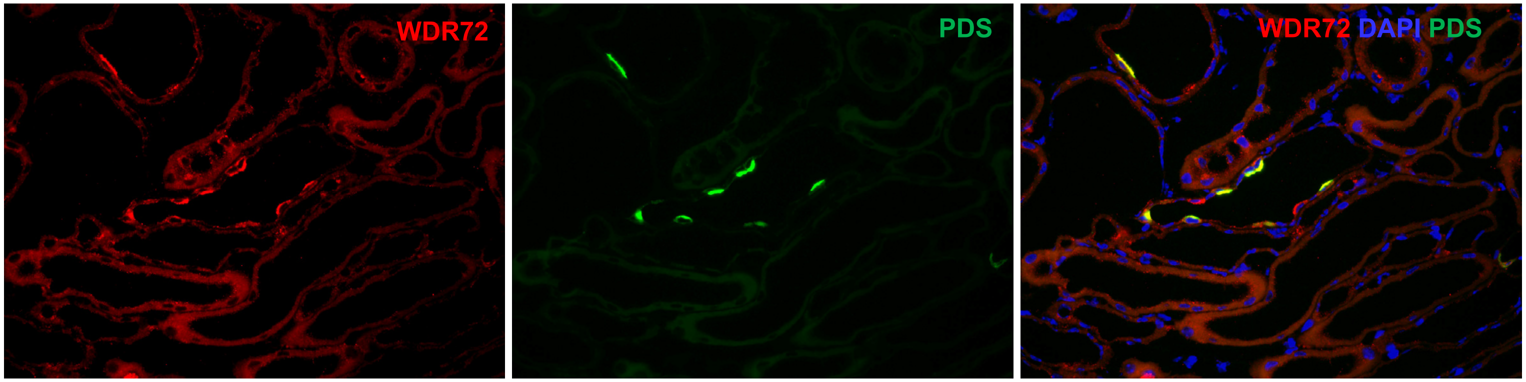

Suppl. Figure 4

Auwerx et al.

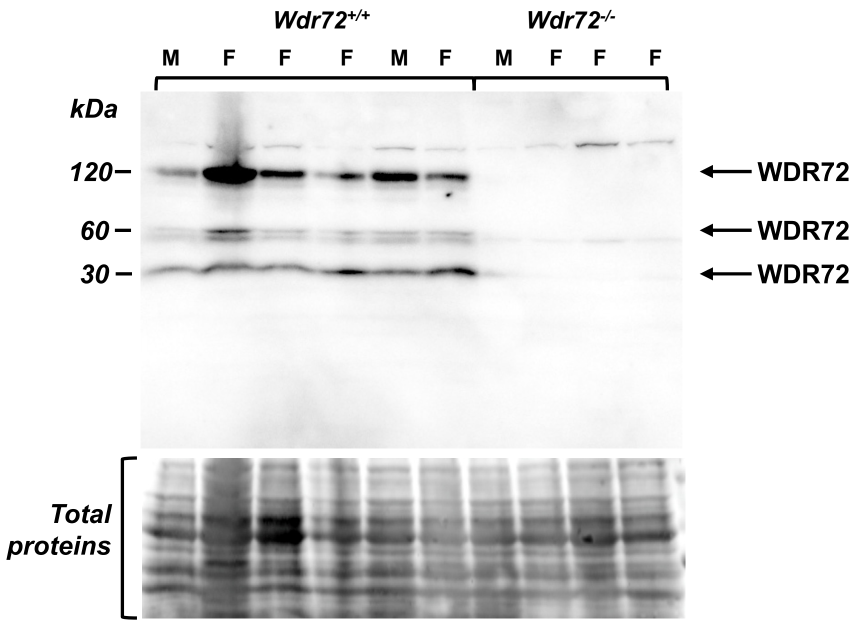

Suppl. Figure 5

Auwerx et al.

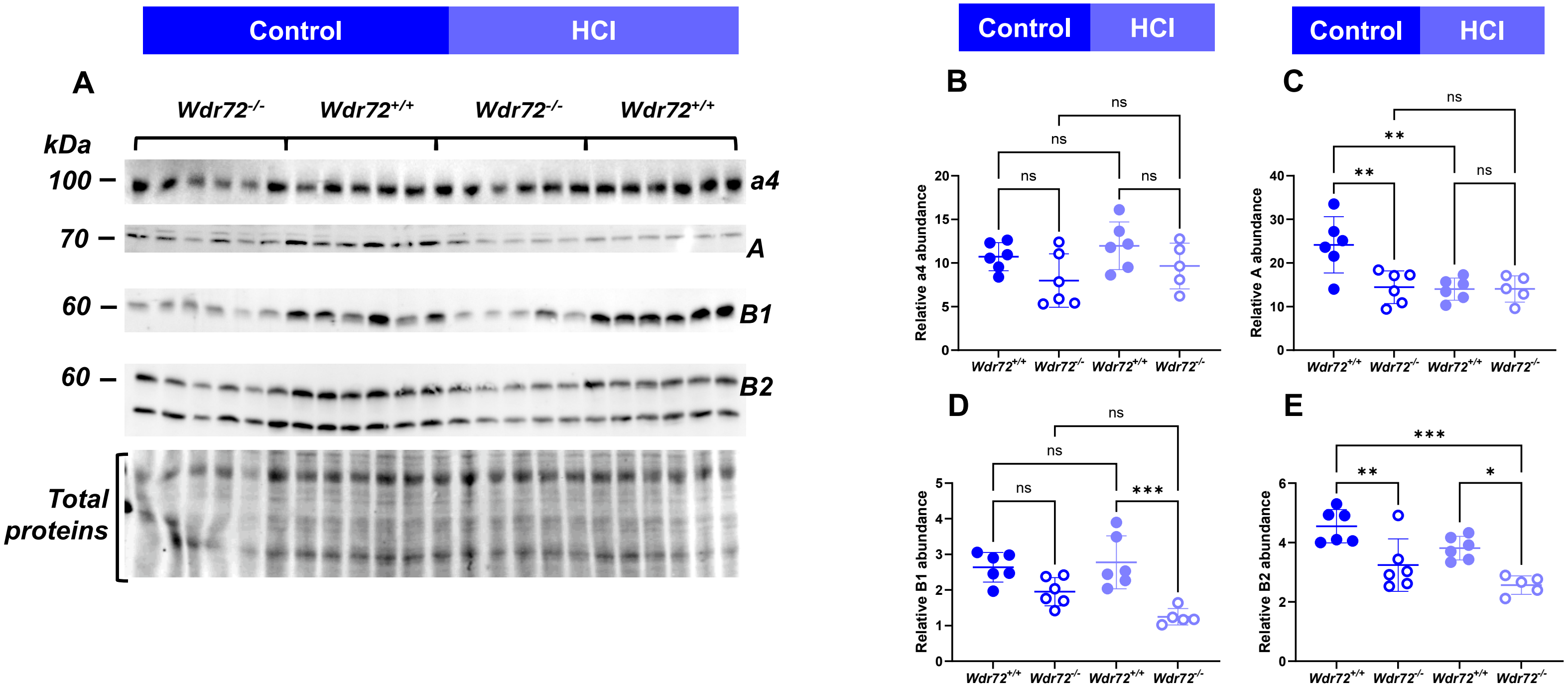

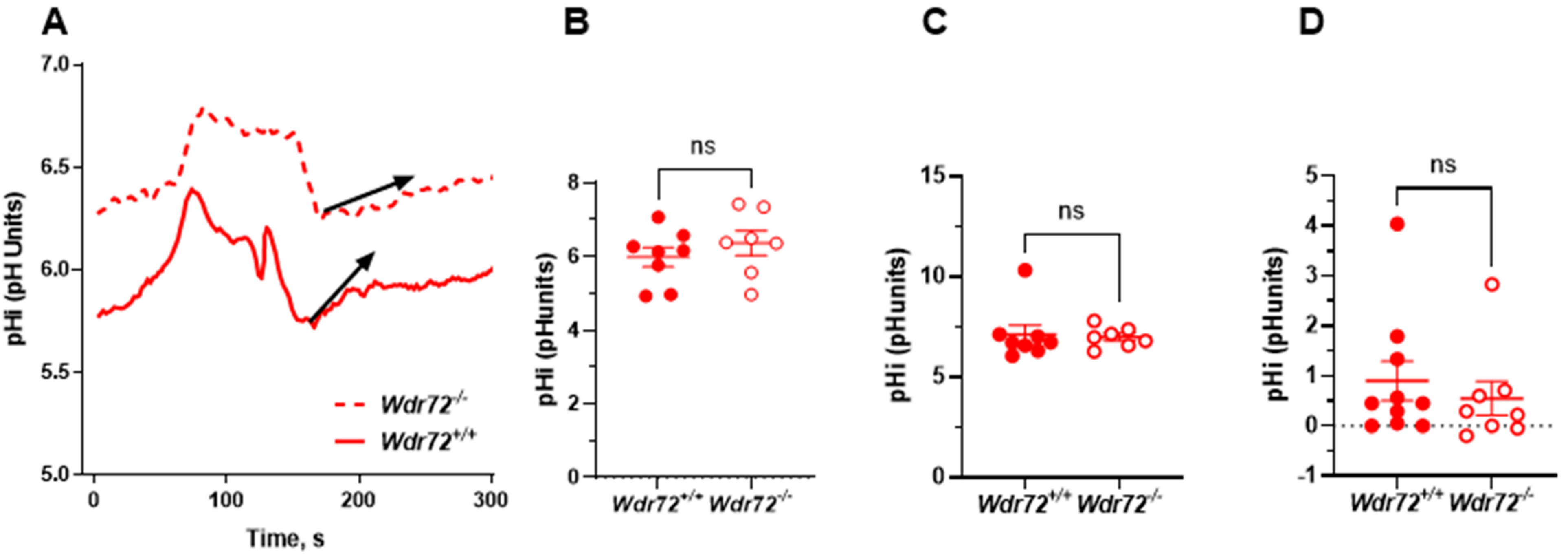
A
7.0
6.5
6.0
5.5
5.0
pHi (pH Units)
0
100
200
300
Time, s
Wdr72-/-
Wdr72+/+
B
ns
8
6
4
2
0
pHi (pHunits)
Wdr72+/+ Wdr72-/-
C
15
10
5
0
ns
pHi (pHunits)
Wdr72+/+ Wdr72-/-
D
5
4
3
2
1
0
-1
ns
pHi (pHunits)
Wdr72+/+ Wdr72-/-

Suppl. Figure 7

Auwerx et al.

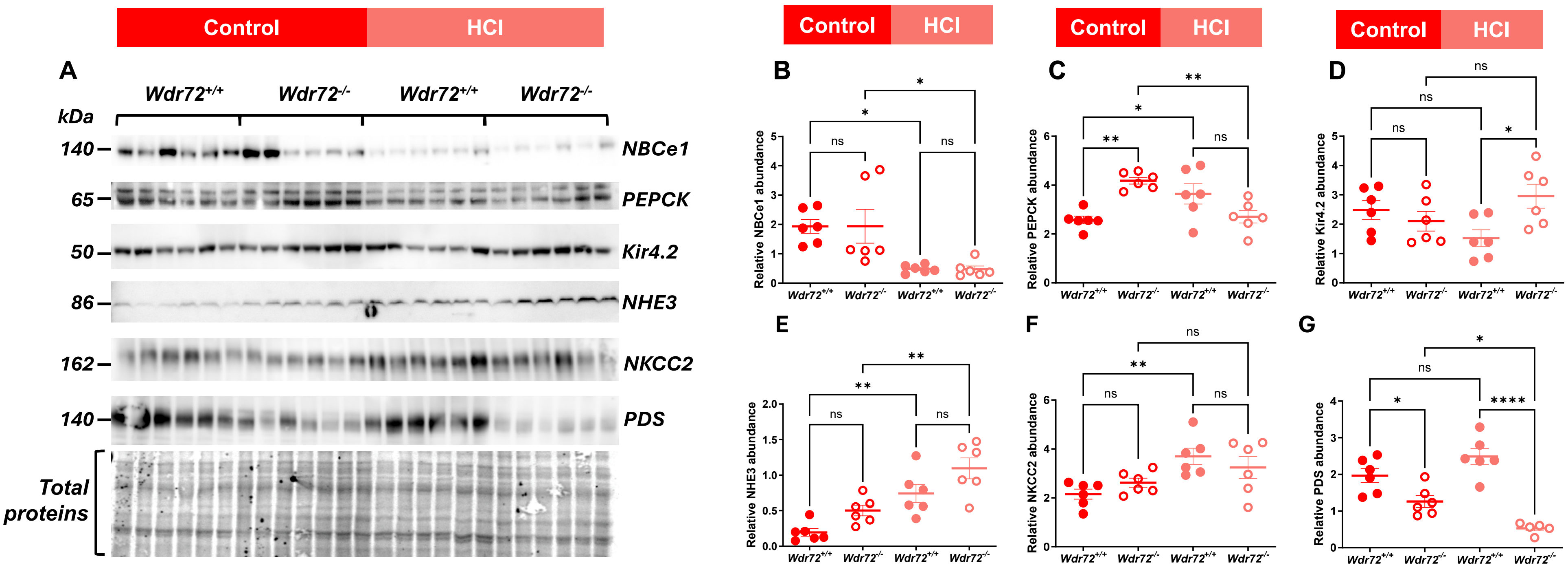

Suppl. Figure 8

Auwerx et al.

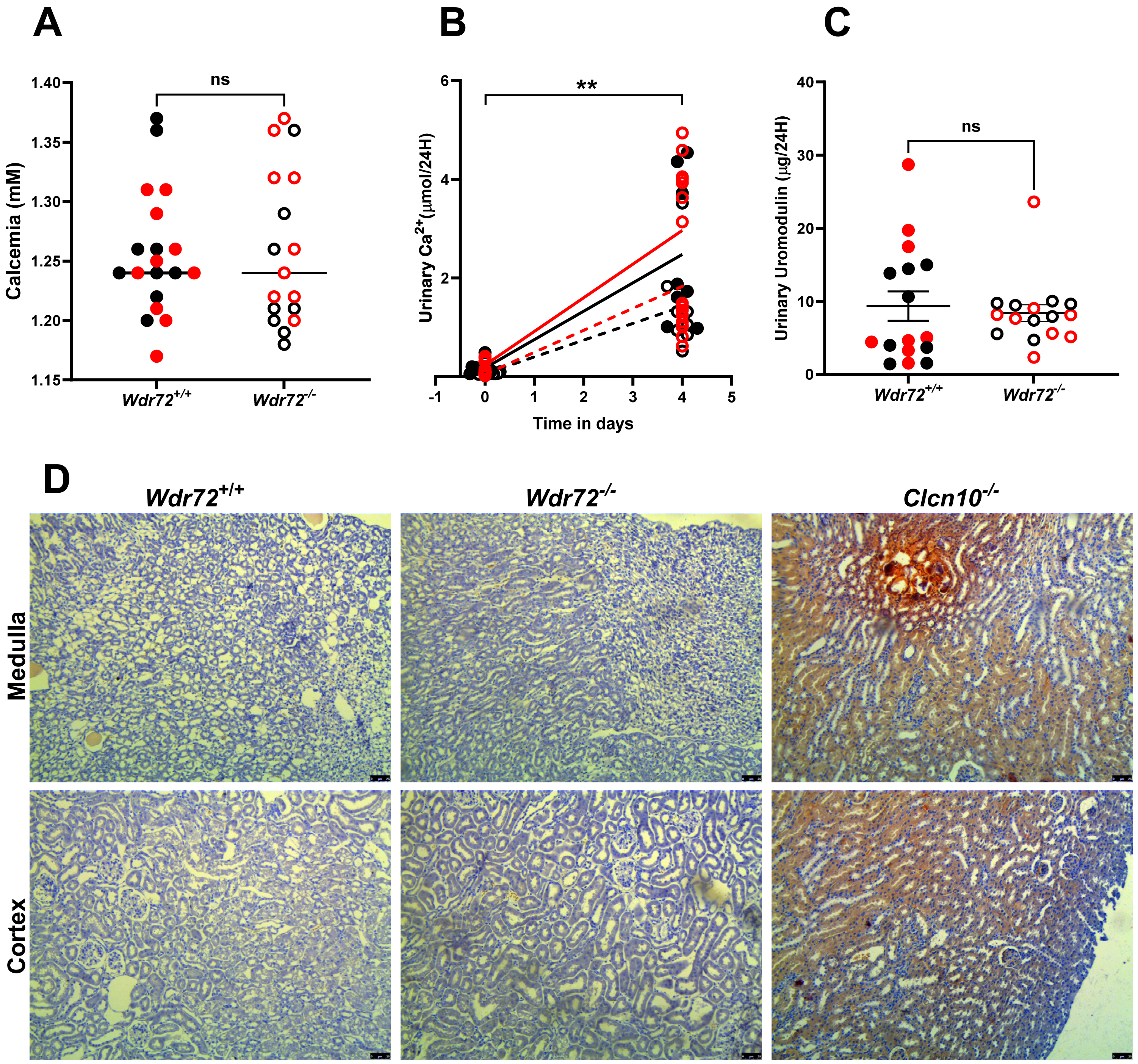

Supplement: Supplementary file 1 — Figure S1: Bright‐field visualization of Wdr72 mRNA expression in mouse renal tubules, including positive and negative controls. Kidney sections from Wdr72 +/+ and Wdr72 −/− mice treated with a 4 days HCl diet. Pictures were acquired on bright‐field microscopy and signal detected as red dots. (A–C) Wdr72 probe on (A and B) Wdr72 +/+ and (C) Wdr72 −/− kidney tissue, (D) negative control probe on Wdr72 +/+ kidney tissue, and (E) positive control probe on Wdr72 −/− kidney tissue. Original magnification 400×. Figure S2: Positive and negative controls of Wdr72 mRNA detection. Kidney sections from Wdr72+/+ and Wdr72−/− mice treated with a 4 days HCl diet, were co‐stained with antibodies against; (A, D and G) NaPiIIa, (B, E and H) NKCC2 or (C, F and I) (all in green) together with the (A–C) Wdr72 probe, (D–F) a positive control probe, or (G–I) a negative control probe. Nuclei were marked in blue using DAPI. (A–C) No Wdr72‐related staining was detected in kidney tissue from Wdr72−/− mice. Original magnification 400×. Figure S3: Immunolocalization of Wdr72 at the apical side of type B intercalated cells from male C57Bl6 wild‐type mouse subjected to 4 days of alkali loading with DOCA/HCO3 −. Mice received deoxycorticosterone (DOCA)/NaHCO₃ treatment via drinking water containing 0.28 M NaHCO₃ and 2% sucrose, along with a single intraperitoneal injection of 2 mg DOCA dissolved in 50 μL DMSO at the start of the 4‐day treatment period. Kidney sections were co‐stained with antibodies against pendrin (PDS, green) and WDR72 (red) together with DAPI in blue. WDR72 was expressed principally at the luminal side of PDS positive cells. Original magnification 400×. Figure S4: Immunoblotting of kidneys from female and male Wdr72+/+ and Wdr72−/− mice. Upper panel: total membrane fractions from female (F) and male (M) Wdr72+/+ and Wdr72−/− mice were tested with an antibody against Wdr72. Three major bands around 120 kDa, 60 kDA, and 30 kDa were detected in kidneys from Wdr72+/+ mice but not [file APHA-242-e70165-s001.pdf]
