# Supplementary material for: WDR72 Is Required for Urinary Acidification and Normal H+‐ATPase Activity in Intercalated Cells in Mice
Source: Acta Physiol (Oxf). 2026 Feb 4;242(3):e70165. doi: 10.1111/apha.70165 (PMC12869471; doi:10.1111/apha.70165)
Supplement: Supplementary file 2 — Table S1: Blood parameters from female and male Wdr72 mice subjected to a control diet. Mean ± SEM, *p ≤ 0.05, **p ≤ 0.01, ***p ≤ 0.00 1 versus Wdr72 +/+ sex‐matched mice. Mean ± SEM, # p ≤ 0.05, ## p ≤ 0.01, ### p ≤ 0.001, #### p ≤ 0.0001 versus same genotype at baseline. In brackets, number of animals. Crea, creatinine; iCa, ionized calcium. In brackets: number of animals. Table S2: Metabolic and urine parameters from control, 2 days and 4 days HCl treated female Wdr72 mice. Values are mean ± SEM, *p < 0.05 versus Wdr72+/+ mice on same diet, # p < 0.05 versus same genotype at baseline, BW, body weight; crea, creatinine. In brackets: number of animals. Table S3: Metabolic and urine parameters from control, 2 days and 4 days HCl treated male Wdr72 mice. Values are mean ± SEM, *p < 0.05 versus Wdr72+/+ mice on same diet, p < 0.05 versus same genotype at baseline, BW, body weight; crea, creatinine. In brackets: number of animals. Table S4: Blood parameters from female and male Wdr72+/+ and Wdr72−/− mice subjected to a HCl diet. Mean ± SEM, *p ≤ 0.05, **p ≤ 0.01, ***p ≤ 0.00 1 versus Wdr72+/+ sex‐matched mice. Mean ± SEM, # p ≤ 0.05, ## p ≤ 0.01, ### p ≤ 0.001, #### p ≤ 0.0001 versus same genotype at baseline. In brackets, number of animals. iCa, ionized calcium; crea, creatinine. In brackets: number of animals. Table S5: Primary antibodies used in this study. Table S6: Composition of microperfusion solutions. [file APHA-242-e70165-s002.pdf]

Suppl. Table 1

Baseline status	Female mice		Male mice	
	Wdr72$^{+/+}$ (N=8)	*Wdr72*$^{-/-}$ (N=7)	*Wdr72*$^{+/+}$ (N=7)	*Wdr72*$^{-/-}$ (N=7)
**Blood values**				
pH	7.41±0.01	7.41±0.02	7.37±0.01	7.37±0.02
pCO_2 (mHg)	31.3±0.8	32.7±1.1	36.2±1.0	36.8±1.1
HCO_3^- (mmol/l)	20.0±0.6	21.0±0.8	21.1±0.5	21.4±0.6
pO_2 (mHg)	58.5±2.7	60.2±5.9	57.9±2.7	64.0±1.9
Cl^- (mmol/l)	114.1±0.3	113.1±1.2	115.6±0.8	114.4±1.3
Na^+ (mmol/l)	145.9±0.3	146.6±0.7	148.4±0.5	147.6±0.8
Urea (mmol/l)	7.7±0.5	7.8±0.7	10.9±1.5	10.2±1.0
Glucose (mmol/l)	12.5±0.7	10.6±0.9	9.9±0.4	11.1±0.9
Lactate (mmol/l)	2.9±0.1	3.9±0.8	3.7±0.4	4.3±0.7
iCa^{2+} (mmol/l)	1.20±0.01	1.19±0.01	1.20±0.01	1.23±0.02
Hematocrit (%)	40.1±0.5	40.0±0.5	40.0±0.6	41.1±0.1
Crea (µmol/l)	*ND*	*ND*	*ND*	*ND*
BE_{ecf} (mmol/l)	-3.5±0.6	-2.9±1.0	-3.6±0.6	-3.3±0.8
K^+ (mmol/l)	4.3±0.2	4.1±0.1	4.8±0.1	4.3±0.1*

Mean ± SEM, In brackets, number of animals. iCa^{2+}: ionized calcium, Crea: Creatinine.

Suppl. Table 2

Female mice	Basal status		2 days HCl in food		4 days HCl in food	
	Wdr72$^{+/+}$ (N=10)	*Wdr72*$^{-/-}$ (N=9)	*Wdr72*$^{+/+}$ (N=10)	*Wdr72*$^{-/-}$ (N=9)	*Wdr72*$^{+/+}$ (N=10)	*Wdr72*$^{-/-}$ (N=9)
**Metabolic values**						
Weight (g)	21.3±0.9	21.0±0.5	20.8±0.7	20.2±0.6	20.0±0.7	19.2±0.6
Food intake (g/24hrs/g BW)	0.40±0.14	0.42±0.12	0.41±0.07	0.34±0.12	0.46±0.06	0.49±0.07
Water intake (ml/24h)	2.0±1.1	1.5±0.8	4.1±2.3	2.5±1.5	4.2±3.7	3.6±1.8
**Urine values**						
Volume (ml/24h)	3.84±0.68	2.65±0.55	4.19±0.56	2.71±0.47	5.08±0.82	4.12±0.49
Creatinine (mmol/l)	9.74±2.26	4.45±0.33*	10.71±1.93	5.37±0.41*	10.84±1.73	7.61±1.10
Crea clearance (ml/min)	ND	ND	ND	ND	0.67±0.11	0.43±0.04
Urea clearance (ml/min)	ND	ND	ND	ND	2.96±0.33	1.41±0.15***
pH	6.3±0.1	6.8±0.1	5.6±0.1	6.2±0.2*	5.8±0.2	6.6±0.3**
NH_4^+ (mEq/24h)	156.0±31.1	78.6±17.2	1734.8±310.7$^{####}$	519.0±49.2***	2241.4±375.5$^{####}$	1028.7±243.2***$^{#}$
Urea (mEq/24h)	3.79±0.66	2.61±0.76	4.43±0.66	2.27±0.342*	4.81±0.63	3.01±0.42*
Na^+ (mEq/24h)	312.9±75.9	153.3±14.9	405.3±72.1	202.9±15.7*	501.2±85.0	315.4±22.6*
Cl^- (mEq/24h)	581.0±102.1	230.0±22.5	2724.0±464.7$^{###}$	890.9±81.9***	3519.0±561.7$^{####}$	1837.0±404.5**$^{#}$
K^+ (mEq/24h)	820.1±150.2	316.7±32.7**	891.2±134.9	386.1±31.5**	1085.8±150.4	606.6±44.0**
Ca^{2+} (mEq/24h)	0.24±0.05	0.05±0.01	ND	ND	2.97±0.61$^{###}$	1.83±0.43*$^{##}$
Uromodulin (µg/24h)	ND	ND	ND	ND	10.62±3.53	8.73±2.26
NGAL (ng/24h)	ND	ND	ND	ND	159.4±38.3	76.7±9.9

Mean ± SEM, *P≤0.05, **P≤0.01, ***P≤0.001 versus Wdr72$^{+/+}$ for sex-matched mice. Mean ± SEM, $^{#}$P≤0.05, $^{##}$P≤0.01, $^{###}$P≤0.001, $^{####}$ P≤0.0001 versus same genotype in basal status. In brackets, number of animals. BW: Body weight, Crea: Creatinine.

Suppl. Table 3

Male mice	Basal status		2 days HCl in food		4 days HCl in food	
	Wdr72$^{+/+}$ (N=9)	*Wdr72*$^{-/-}$ (N=9)	*Wdr72*$^{+/+}$ (N=9)	*Wdr72*$^{-/-}$ (N=9)	*Wdr72*$^{+/+}$ (N=9)	*Wdr72*$^{-/-}$ (N=9)
**Metabolic values**						
Weight (g)	27.3±0.7	27.2±1.0	25.7±0.6	25.6±0.8	25.3±0.5	25.2±0.8
Food intake (g/24hrs/g BW)	0.26±0.11	0.30±0.10	0.31±0.10	0.32±0.06	0.37±0.04$^{\#}$	0.38±0.06
Water intake (ml/24h)	1.5±0.9	2.9±2.4	3.8±2.7	5.0±2.5	4.8±4.4	5.9±3.2
**Urine values**						
Volume (ml/24h)	2.91±0.54	3.27±0.39	3.56±0.65	3.64±0.54	4.44±0.64	4.31±0.34
Creatinine (mmol/l)	7.11±1.48	7.27±0.42	6.60±0.57	7.24±0.62	10.15±1.68	9.48±1.89
Crea clearance (ml/min)	ND	ND	ND	ND	0.56±0.11	0.67±0.14
Urea clearance (ml/min)	ND	ND	ND	ND	1.65±0.27	1.41±0.22
pH	6.2±0.1	6.7±0.1*	5.6±0.2	6.1±0.1*	5.6±0.2	6.5±0.3***
NH_4^+ (mEq/24h)	72.1±9.7	107.0±32.9	538.5±62.7$^{\#}$	731.8±178.6	902.5±250.4$^{\#\#\#\#}$	1412.6±333.6$^{\#\#}$
Urea (mEq/24h)	2.81±0.68	2.76±0.52	2.49±0.37	2.58±0.29	3.77±0.52	3.41±0.65
Na^+ (mEq/24h)	188.6±44.3	225.6±22.1	236.1±33.8	405.3±72.1	416.9±71.6$^{\#\#}$	501.2±85.0
Cl^- (mEq/24h)	351.4±89.3	394.2±66.7	1334.1±248.3	1043.0±99.7	2489.2±500.8$^{\#\#\#\#}$	1735.5±400.9$^{\#\#}$
K^+ (mEq/24h)	553.7±138.8	558.6±100.3	526.1±83.1	515.4±53.4	857.5±127.0	720.7±120.43
Ca^{2+} (mEq/24h)	0.18±0.05	0.06±0.01	ND	ND	2.48±0.53$^{\#\#\#\#}$	1.42±0.33*$^{\#\#}$
Uromodulin (µg/24H)	ND	ND	ND	ND	8.09±2.11	8.08±0.72
NGAL (ng/24h)	ND	ND	ND	ND	157.1±63.0	97.4±15.0

Mean ± SEM, *P≤0.05, **P≤0.01, ***P≤0.001 versus Wdr72$^{+/+}$ for sex-matched mice. Mean ± SEM, $^{\#}$P≤0.05, $^{\#\#}$P≤0.01, $^{\#\#\#}$P≤0.001, $^{\#\#\#\#}$ P≤0.0001 versus same genotype in basal status. In brackets, number of animals. BW: Body weight, Crea: Creatinine.

Suppl. Table 4

4 days HCl in food	Female mice		Male mice	
	Wdr72$^{+/+}$ (N=10)	*Wdr72*$^{-/-}$ (N=9)	*Wdr72*$^{+/+}$ (N=9)	*Wdr72*$^{-/-}$ (N=8)
**Blood values**				
pH	7.37±0.02	7.27±0.03**	7.31±0.02	7.30±0.03
pCO_2 (mHg)	31.7±0.8	32.3±0.9	36.1±1.0	35.4±1.1
HCO_3^- (mmol/l)	18.6±0.8	15.0±1.0*	18.4±1.0	17.5±1.2
pO_2 (mHg)	55.0±1.8	63.5±2.7*	58.8±3.1	58.8±3.1
Cl^- (mmol/l)	118.6±0.7	123.0±1.9*	121.2±1.2	120.3±2.1
Na^+ (mmol/l)	149.3±0.4	150.8±1.1	149.8±0.7	149.9±1.2
Urea (mmol/l)	6.7±0.5	9.1±0.8*	9.35±0.6	9.8±0.8
Glucose (mmol/l)	10.0±0.5	9.9±0.9	8.9±0.8	10.5±1.0
Lactate (mmol/l)	2.8±0.3	3.9±0.3*	3.0±0.3	4.9±0.9
iCa^{2+} (mmol/l)	1.25±0.01	1.28±0.02	1.27±0.02	1.24±0.02
Hematocrit (%)	37.9±1.3	38.2±0.5	39.3±0.8	39.1±0.5
Crea (µmol/l)	0.13±0.00	0.14±0.01	0.13±0.01	0.13±0.02
BE_{ecf} (mmol/l)	-6.6±1.1	-11.1±1.4*	-7.8±1.3	-9.1±1.7
K^+ (mmol/l)	4.5±0.1	4.4±0.1	4.8±0.1	4.5±0.2

Mean ± SEM, *P≤0.05, **P≤0.01, ***P≤0.001 versus Wdr72$^{+/+}$ for sex-matched mice. Mean ± SEM, $^{\#}$P≤0.05, $^{\#\#}$P≤0.01, $^{\#\#\#}$P≤0.001, $^{\#\#\#\#}$ P≤0.0001 versus same genotype in basal status. In brackets, number of animals. iCa^{2+}: ionized calcium, Crea: Creatinine.

Suppl. Table 5

	Name	Manufacturer	Working dilution	Catalog Number or reference
Immunohistochemistry	Rabbit anti-NaPiIIa	Homemade	1:1000	[1]
	Rabbit anti-NKCC2		1:1000	a kind gift from Johannes Loffing, Institute of Anatomy, University of Zurich
	Guinea Pig anti-AE1	Homemade	1:500	[2]
	Rabbit anti-WDR72	Sigma Aldrich, CH	1:1000	HPA048212
	Rabbit anti-RhCG		1:1000	a kind gift from Yves Colin, INSERM, Paris, France [3]
	Rabbit anti-a4	Homemade	1:1000	[4]
	Rabbit anti-B1	Homemade	1:1000	[5]
	Rabbit anti-B2	Homemade	1:1500	[6,7]
	Rabbit anti-A	Homemade	1:400	[6,7]
	Rabbit anti-G3	Abcam, UK	1:200	ab122012
Immunoblot	Rabbit anti-WDR72	Sigma Aldrich, CH	1:1000	HPA048212
	Rabbit anti-a4	Homemade	1:2000	[4]
	Rabbit anti-A	Homemade	1:2000	[6,7]
	Rabbit anti-B1	Homemade	1:5000	[5]
	Rabbit anti-B2	Homemade	1:5000	[6,7]
	Rabbit anti G3	Abcam, UK	1:1000	Ab122012
	Rabbit anti-PEPCK	Cayman Chemical, USA	1:5000	ref10004943
	Rabbit anti-NKCC2		1:5000	a kind gift from Johannes Loffing, Institute of Anatomy, University of Zurich
	Rabbit anti-PDS	Homemade	1:2000	[8]
	Rabbit anti-NBCe1	Proteintech, UK	1:5000	11885-1AP
	Rabbit anti-Kir4.2	Alomone Labs, Israel	1:200	APC-058
	Rabbit anti-NHE3	StressMarq Biosciences Inc.	1:2000	SPC-400D

Suppl. Table 6

Components	Solutions			
	**A Dissection solution**	**B Calibration solution**	**C 0 Cl^- 0 NH_4^+**	**D 0 Cl-, 20 NH_4^+**
Na^+	138	15	0	0
K^+	4	90	4	4
$NMDG^+$	0	30	136	116
Ca^{2+}	1.5	0	1.5	1.5
Mg^{2+}	1.2	1.5	1.2	1.2
NH_4^+	0	0	0	20
**meq (+)**	**144.7**	**136.5**	**142.7**	**142.7**
Cl^-	141.5	134	139.5	139.5
HCO_3^-	0	0	0	0
HPO_4^{2-}	2	2.5	2	2
SO_4^{2-}	1.2	0	1.2	1.2
$Gluconate^-$	0	0	0	0
**meq (−)**	**144.7**	**136.5**	**142.7**	**142.7**
Glucose	5.5	0	5.5	5.5
Alanine	5	0	0	0
HEPES	10	25	10	10
pH	7.4		7.4	7.4

All concentrations are given in mmol/l

References for supplementary data

1. Custer M, Lotscher M, Biber J, Murer H, Kaissling B. Expression of Na-P(i) cotransport in rat kidney: localization by RT-PCR and immol/lunohistochemistry. *Am J Physiol*. May 1994;266(5 Pt 2):F767-774. doi:10.1152/ajprenal.1994.266.5.F767
2. Stehberger PA, Shmukler BE, Stuart-Tilley AK, Peters LL, Alper SL, Wagner CA. Distal renal tubular acidosis in mice lacking the AE1 (band3) Cl-/HCO3- exchanger (slc4a1). *J Am Soc Nephrol*. May 2007;18(5):1408-1418. doi:10.1681/ASN.2006101072
3. Eladari D, Cheval L, Quentin F, et al. Expression of RhCG, a new putative NH(3)/NH(4)(+) transporter, along the rat nephron. *J Am Soc Nephrol*. Aug 2002;13(8):1999-2008. doi:10.1097/01.asn.0000025280.02386.9d
4. Stehberger PA, Schulz N, Finberg KE, et al. Localization and regulation of the ATP6V0A4 (a4) vacuolar H+-ATPase subunit defective in an inherited form of distal renal tubular acidosis. *J Am Soc Nephrol*. Dec 2003;14(12):3027-3038. doi:10.1097/01.asn.0000099375.74789.ab
5. Wagner CA, Lukewille U, Valles P, et al. A rapid enzymatic method for the isolation of defined kidney tubule fragments from mouse. *Pflugers Arch*. Aug 2003;446(5):623-632. doi:10.1007/s00424-003-1082-3
6. Pathare G, Dhayat N, Mohebbi N, et al. Change in V-ATPase B1 but not B2 subunit abundance in human urinary exosomes in response to acute acid/alkali loading and distal renal tubular acidosis. *Swiss Med Wkly*. Dec 4 2017;147:11s-11s.
7. Pathare G, Dhayat NA, Mohebbi N, et al. Changes in V-ATPase subunits of human urinary exosomes reflect the renal response to acute acid/alkali loading and the defects in distal renal tubular acidosis. *Kidney International*. Apr 2018;93(4):871-880. doi:10.1016/j.kint.2017.10.018
8. Hafner P, Grimaldi R, Capuano P, Capasso G, Wagner CA. Pendrin in the mouse kidney is primarily regulated by Cl- excretion but also by systemic metabolic acidosis. *Am J Physiol Cell Physiol*. Dec 2008;295(6):C1658-1667. doi:10.1152/ajpcell.00419.2008
